# A deuterostome origin of the Spemann organiser suggested by Nodal and ADMPs functions in Echinoderms

**DOI:** 10.1038/ncomms9434

**Published:** 2015-10-01

**Authors:** François Lapraz, Emmanuel Haillot, Thierry Lepage

**Affiliations:** 1 Institut de Biologie Valrose, iBV, UMR 7277 CNRS, Inserm U1091, UNS, University of Nice Sophia Antipolis Nice 06108, 2 France

## Abstract

During development of chordates, establishment of the body plan relies on the activity of an organizing centre located on the dorsal side of the embryo that patterns the embryo and induces neural tissue. Intriguingly, the evolutionary origin of this crucial signalling centre remains unclear and whether analogous organizers regulate D/V patterning in other deuterostome or protostome phyla is not known. Here we provide evidence that the ventral ectoderm of the sea urchin embryo is a long-range organizing centre that shares several fundamental properties with the Spemann organizer: the ability to induce duplicated embryonic axes when ectopically induced, the ability to induce neural fate in neighbouring tissues and the ability to finely regulate the level of BMP signalling by using an autoregulatory expansion–repression mechanism. These findings suggest that the evolutionary origin of the Spemann organizer is more ancient than previously thought and that it may possibly be traced back to the common ancestor of deuterostomes.

## Abstract (web summary)

Establishment of the body plan in chordates is determined by an organizing centre located on the dorsal side of the embryo. Here, the authors show that the ventral ectoderm of the sea urchin embryo is an organizing centre that shares several fundamental properties with the amphibian Spemann organizer.

## Graphical abstract

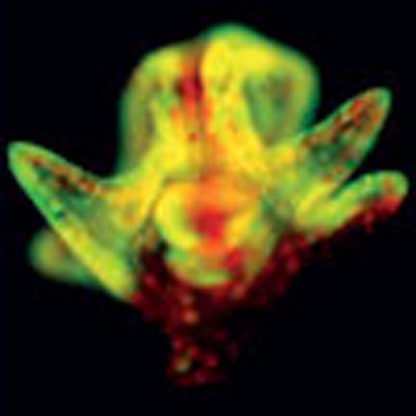

The Spemann organizer was first identified as a group of cells that can induce development of Siamese twins when transplanted^1^,^2^,^3^. We now know that in order to induce a nervous system and to pattern the embryo the organizer secretes a cocktail of bone morphogenetic proteins (BMP) and Wnt antagonists such as Chordin, Noggin and Frzb, that are produced downstream of Nodal and β-catenin and that counteract the ventralizing activity of BMP and Wnt ligands produced ventrally^4^,^5^,^6^. In addition to possessing an organizing activity and to induce neural tissue, the Spemann organizer possesses another remarkable property: it is capable of self-regulation^2^,^3^. The striking ability of the Spemann organizer to self-regulate was recently shown to rely on the secretion by the organizer of an atypical BMP ligand called ADMP (ref. 7). Unlike *bmp4*, the expression of which is activated by BMP signalling, *admp* is repressed by BMP signalling^7^,^8^,^9^. When BMP signalling goes down, expression of *admp* goes up and ADMP protein is shuttled by Chordin to the ventral side where it promotes BMP signalling and expression of *bmp4*. This simple design based on two BMP ligands expressed at opposite poles under opposite transcriptional control and that can both be shuttled by Chordin is thought to participate to D/V patterning^10^,^11^,^12^ and to provide robustness to fluctuations of BMP signalling along the D/V axis^3^,^7^,^13^,^14^.

*admp* genes are present in the genome of most bilaterians including non-chordate deuterostomes such as hemichordates^15^ and echinoderms^16^ as well as in lophotrocozoa^17^, but absent from many ecdysozoa^18^. However, the function of these BMP-ADMP circuits have been studied so far only during organizer function in chordates and during regeneration in the adult in planarians^18^,^19^ and acoels^20^.

In echinoderms like in chordates, D/V axis formation relies on the activity of the TGF-β Nodal. The mechanisms that establish the spatial restriction of *nodal* expression are not well understood^21^,^22^,^23^,^24^,^25^,^26^. The current prevailing model postulates that redox gradients generated by mitochondria asymmetrically distributed in the egg regulate the activity of redox sensitive transcription factors that control the initial asymmetry of *nodal* expression^21^,^27^. However, although very attracting, the hypothesis that mitochondrial redox gradients drive *nodal* expression is not strongly supported by the extensive experimental work that has addressed this question. Recently, the homeobox transcription factor Hbox12, a member of the *pmar1*/*hbox12*/*micro1* family has been proposed to regulate the early expression of *nodal*^26^, however, conflicting results were subsequently reported regarding the expression pattern and activity of this gene, questioning the idea that *hbox12* plays a role in the regulation of *nodal* expression^22^. Remarkably, both in echinoderms and in chordates, Wnt and Univin/Vg1 signalling are required for *nodal* expression^24^,^27^,^28^. Univin/Vg1 is required for high level of Nodal signalling and for maintaining the Nodal autoregulatory loop^27^. Canonical Wnt signalling is also thought to be required to maintain the *nodal* autoregulatory loop^24^,^27^ and ligands such as Wnt1 and Wnt8 have been proposed to regulate *nodal* expression through respecification and patterning of the ectoderm and non canonical signalling^29^. Wnt1 has also been proposed to limit *nodal* expression in the vegetal pole region^25^ but the functional significance of this restriction of *nodal* expression is unclear since ectopic activation of Nodal signalling in the vegetal pole region has no consequence on patterning of the embryo and Nodal appears instead to be required for patterning of the vegetal ectoderm^30^.

Unlike in chordates, where *nodal* is expressed dorsally, in the sea urchin, *nodal* is expressed ventrally^31^, consistent with a hypothetical inversion of the D/V axis having occurred in the chordate lineage. Nodal expression is essential for D/V patterning and knocking-down *nodal* with a morpholino eliminates D/V polarity, resulting in embryos that are fully radialized and lack a mouth. However, injection of *nodal* mRNA into one blastomere of *nodal* morphants is sufficient to completely rescue D/V polarity and to reorganize the pattern over a long range. This suggests that Nodal expressing cells have a large-scale organizing activity that is reminiscent of the long-range organizing activity of the Spemann organizer in amphibians^31^. Although there are functional similarities between the ventral ectoderm of the sea urchin embryo and the Spemann organizer, whether the Nodal-expressing region of the sea urchin embryo really defines a D/V organizer and whether it can induce duplicated D/V axes including a nervous system when created ectopically has never been shown experimentally. Similarly, it is not known if the self-regulatory BMP-ADMP circuit that has been implicated in the auto-adjustment of BMP signalling and in scaling in chordates also operates in the sea urchin embryo since intriguingly, in the sea urchin, BMP2/4 does not autoregulate. Furthermore, *admp2*, the only *ADMP* gene that has been characterized functionally so far, is expressed on the opposite side of the source of BMP2/4, and unlike ADMP in chordates, its transcription is activated, not repressed, by BMP signalling.

In this study, we provide evidence that the Nodal expressing territory of the sea urchin embryo is functionally equivalent to the Spemann organizer with regard to its ability to induce a duplicated and fully patterned D/V axis when ectopically created and regarding its ability to induce neural tissue. We also show that an ADMP based autoregulatory circuit functions in this organizer to regulate cell fate specification along the D/V axis. Taken together, these findings suggest that a signalling centre functionally equivalent to the Spemann organizer was already present in the common ancestor of deuterotomes.

## Results

### Alk4/5/7QD induces mirror-image duplications of the D/V axis

**Figure 1:**
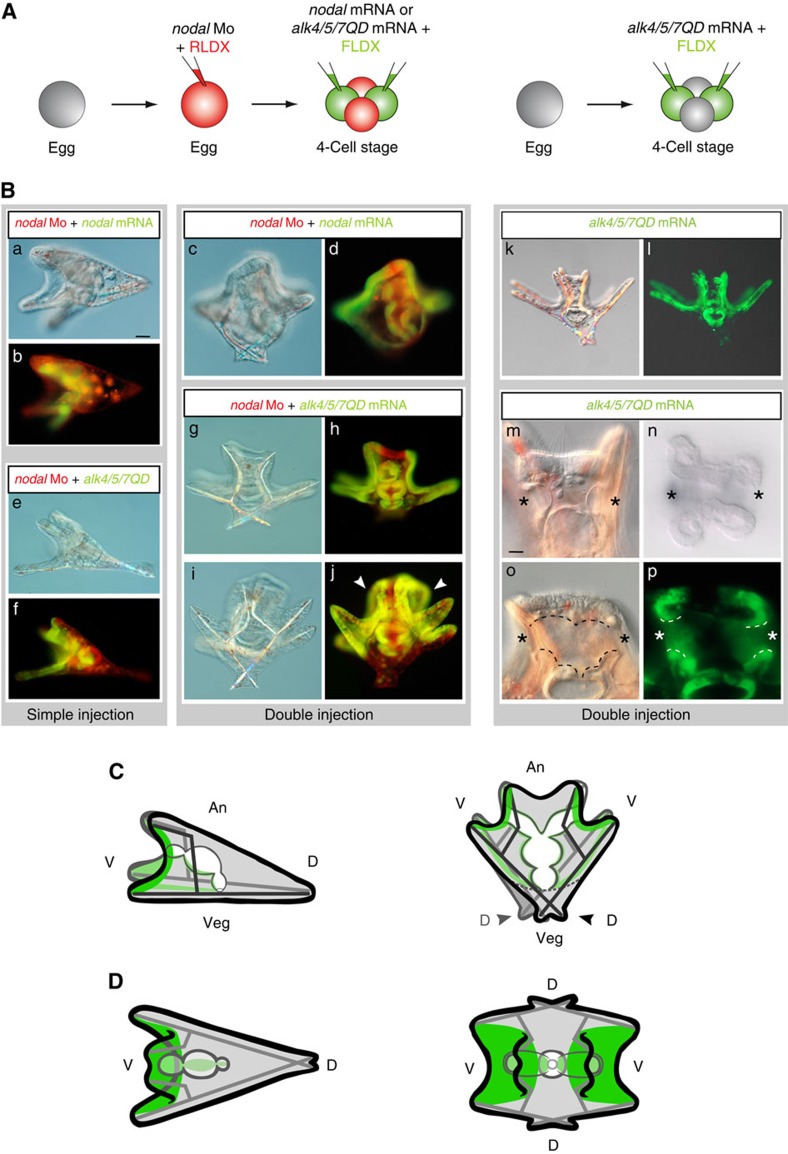
Ectopic Nodal generates Siamese pluteus larvae with two skeletons, two oral lobes, two mouths, two dorsal sides and two ciliary bands. (**A**) Eggs injected with a *nodal* morpholino and RLDX were re-injected at the four-cell stage, into one randomly chosen or into two opposite blastomeres with either mRNA encoding the diffusible ligand Nodal or the Alk4/5/7QD activated Nodal receptor together with FLDX as a lineage tracer. (**B**) Phenotypes induced by a single or double source of Nodal signalling in a *nodal* morphant background. (a,b,e,f) Typical morphology of 72 h rescued embryos derived from a single injection of *nodal* (a,b) or *alk4/5/7QD* (e,f) mRNA. Note that the progeny of the injected cell contributes exclusively to the ventral ectoderm, mesoderm and gut. (c,d,g–j) Lateral views of 72 h embryos doubly injected with either mRNA encoding Nodal or the activated Nodal receptor together with FLDX. (c,d) In the case of *nodal*, the progeny of the injected blastomeres (green) are facing in opposite directions each forming two short oral arms and an oral lobe. The oral lobes derived from each injection clone are fused back to back in the animal-most region formed of ciliary band-like ectoderm. Two pairs of spicules are present in these embryos. (g–j) Two embryos (g,h,i,j) doubly injected with the a*lk4/5/7QD* mRNA. The larvae are viewed as in **C**. The two ventral sides are respectively on the left and right sides of the picture. Note the two well visible ciliary bands in the embryo in j (white arrows). (k–p) Siamese larvae derived from a double injection of *alk4/5/7QD* mRNA into two opposite blastomeres at the four-cell stage (without prior injection of the Nodal morpholino). The close up in m–p show the two stomodeal invaginations before (m) or after (n–p) fusion with the tip of the gut. (**C**,**D**) Schemes depicting lateral views (**C**) or animal pole views (**D**) of *nodal* morphants rescued by a single (left) or double (right) injection of *nodal* or *alk4/5/7QD* mRNA. The progeny of injection clones is highlighted in green. Note that instead of converging towards a unique dorsal apex, the two spicules that originate from a given ventral side diverge to join two different dorsal apex. Scale bar, 20 μm in a–l; and 5 μm in m–p. FLDX, Fluoresceinated Dextran; RLDX, Rhodamine Dextran.

**Figure 2:**
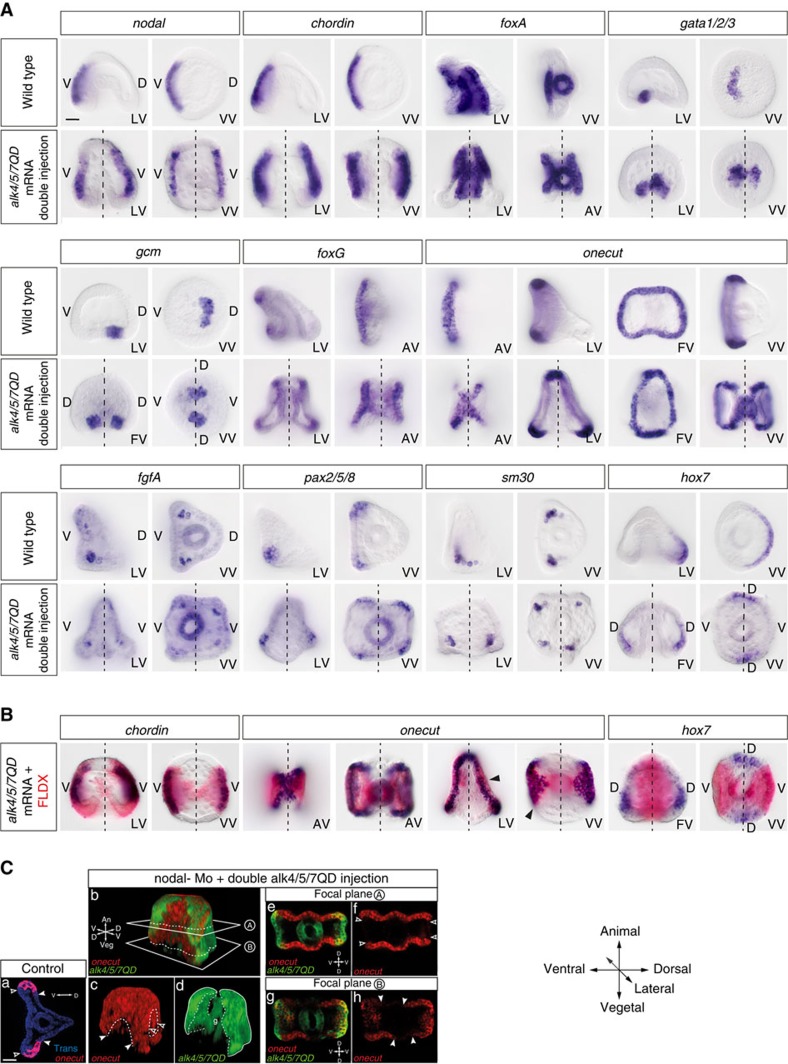
Ectopic Nodal respecifies the ectoderm, induces ectopic neural tissues and reorganizes patterning of the skeleton and secondary mesoderm. (**A**) Double injection of *alk4/5/7QD* mRNA duplicated the territories expressing *nodal*, *chordin*, *foxA* and induced ectopic territories expressing the PMC cluster marker *sm30* and the lateral ectoderm markers *pax2/5/8*, and *fgfA*. It also duplicated the dorsal territory expressing *hox7* and the ventral and dorsal secondary mesodermal lineages expressing *gata1/2/3* and *gcm*. Finally, ectopic *alk4/5/7QD* triggered formation of two sharply delimited ciliary bands that expressed *foxG* and *onecut*. (**B**) Cell lineage analysis of embryos doubly injected with *alk4/5/7QD* mRNA. In situ hybridization (dark blue) of *chordin*, *onecut* and *hox7*. The red color identifies the clone of *alk4/5/7QD* expressing cells. Note that while *chordin* is induced within the clone of injection, *hox7* instead is induced non-autonomously, at 90 °C of the clone of injected cells. Note that the belts of cells expressing *onecut* partially overlap with the clone of injected cells in most of the ciliary band. Also note the almost perfect congruence of the *alk4/5/7QD* expressing territory and *onecut* expressing territory in the vegetal pole region. (**C**) Confocal observation of embryos following fluorescent in situ hybridizations with the ciliary band marker *onecut* in control and double axis gastrulae. (a) Control embryo seen from the animal pole express *onecut* in a ring of thick cells around the ventral ectoderm. *onecut* is shown in red, outline of the embryo observed using transmission is in blue. *onecut* expression is absent in the ventral ectoderm (black arrows) and the dorsal ectoderm (white arrows). (b–d) Three-dimensional reconstruction of a *nodal* morphant gastrula obtained by double injection of *alk4/5/7QD*. red, *onecut* expression. green, progeny of the *alk4/5/7QD* injected clones. g, gut. (e,f) *onecut* (red), as seen on the focal plane A (see b), is expressed in the whole ectoderm, but is absent in two regions (black arrows) corresponding to the progeny of injected blastomere (green) (e). (g,h) *onecut*, as seen on the vegetal focal plane B (see b), is expressed through most of the ectoderm, but is absent from two regions (white arrows) found in between the progeny of the injected clones (green) that correspond to the presumptive dorsal ectoderm (g). Scale bar, 20 μm in A–C.

Embryos injected with the Nodal morpholino then doubly injected with either *nodal* mRNA or mRNA encoding an activated Nodal receptor developed into Siamese twins larvae fused back to back, with one gut but with two pairs of harmoniously patterned spicules, two oral lobes and two pairs of well elongated ventral arms, (Fig. 1A,B) (*n*>300). In these embryos, although the archenteron remained straight at the centre of the larva, two stomodeal invaginations usually formed and later fused with the tip of the archenteron, resulting in larvae with two mouths opening into a single oesophagus (Fig. 1Bm–p). In addition to these duplicated ventral structures, two belts of thick cuboidal cells surrounding the oral lobes that looked like duplications of the ciliary band (Fig. 1Bg–j) and two dorsal apex (Fig. 1Bc,g,i,k) made of a thin squamous epithelium formed in these larvae. As in the case of *nodal* morphants rescued by single injection of *nodal* mRNA, the progeny of the injected cells only contributed to the oral regions of these embryos, consistent with the non-autonomous action of Nodal and Alk4/5/7QD on specification of the dorsal side^30^,^31^. Molecular analysis of Siamese embryos revealed the presence at gastrula stage of two opposing territories expressing *nodal*, *chordin* and *foxA* and of four clusters of primary mesenchymal cells (PMCs) expressing the PMC cluster marker genes SM30 and FGFA (Fig. 2A). Indeed, marker genes of the lateral ectoderm, which produces signals that guide migration and clustering of the PMCs, including FGFA and *pax2/5/8* were expressed in four discrete regions in the doubly injected embryos instead of two in the controls. Similarly, two territories facing each other and expressing the dorsal marker gene *hox7* were present in embryos doubly injected with *alk4/5/7QD* indicating that two presumptive dorsal sides had been specified. Intriguingly, lineage labelling revealed that while Alk4/5/7QD induced *chordin* expression cell autonomously, misexpression of the activated receptor induced *hox7* expression non-autonomously in regions located orthogonal to the clones (Fig. 2B). Finally, duplicated territories expressing the ventral and dorsal mesodermal marker genes *gata1/2/3* and *gcm* were present in the vegetal pole region of the Siamese larvae. So not only ectopic activation of Nodal signalling can rescue an axis but it can induce and coherently pattern a full ectopic D/V axis with dorsal and ventral sides, consistent with the idea that Nodal expressing cells are indeed a D/V organizer.

### Ectopic Nodal signalling induces ectopic ciliary bands

A characteristic feature of the Spemann organizer is that it induces and patterns neural tissue by producing BMP antagonists, which in turn are responsible for neural induction through BMP inhibition^5^. We therefore analysed the expression of the ciliary band marker genes *onecut*/*hnf6* and *foxG*, two emblematic neural markers expressed in nested territories at the border of the *nodal* expressing ventral ectoderm, in these Siamese larvae (Fig. 2C)^32^,^33^,^34^,^35^,^36^,^37^. In gastrulae containing two opposite sources of Nodal signalling, *onecut* and *foxG* were expressed in two continuous and sharply delimited belts of cuboidal cells facing one another that included the vegetal most and lateral ectoderm and that gathered at the level of the animal pole region (Fig. 2A). Lineage labelling revealed that these belts of *onecut*-positive cells encircled and partially overlapped with the clones of *alk4/5/7QD* expressing cells (Fig. 2B). Furthermore, in the vegetal pole region the territory of *onecut* positive cells precisely overlapped with the clone, strongly suggesting that at least a subdomain of these induced ciliary bands had been induced as a direct consequence of ectopic Nodal signalling. Confocal analysis confirmed that *onecut* was broadly expressed in territories located between and at the periphery of the clones (Fig. 2Ca–f) but absent in two regions found more vegetally in between the injection clones (Fig. 2Cg–h; Supplementary movies 1, 2 and 3 that likely corresponded to the presumptive dorsal regions (see also below). We conclude that in the sea urchin embryo, like in chordates, ectopic activation of Nodal signalling either directly or indirectly, induces and patterns neural tissue.

### Shuttling of BMP2/4 reorganizes the D/V axis of Siamese embryos

**Figure 3:**
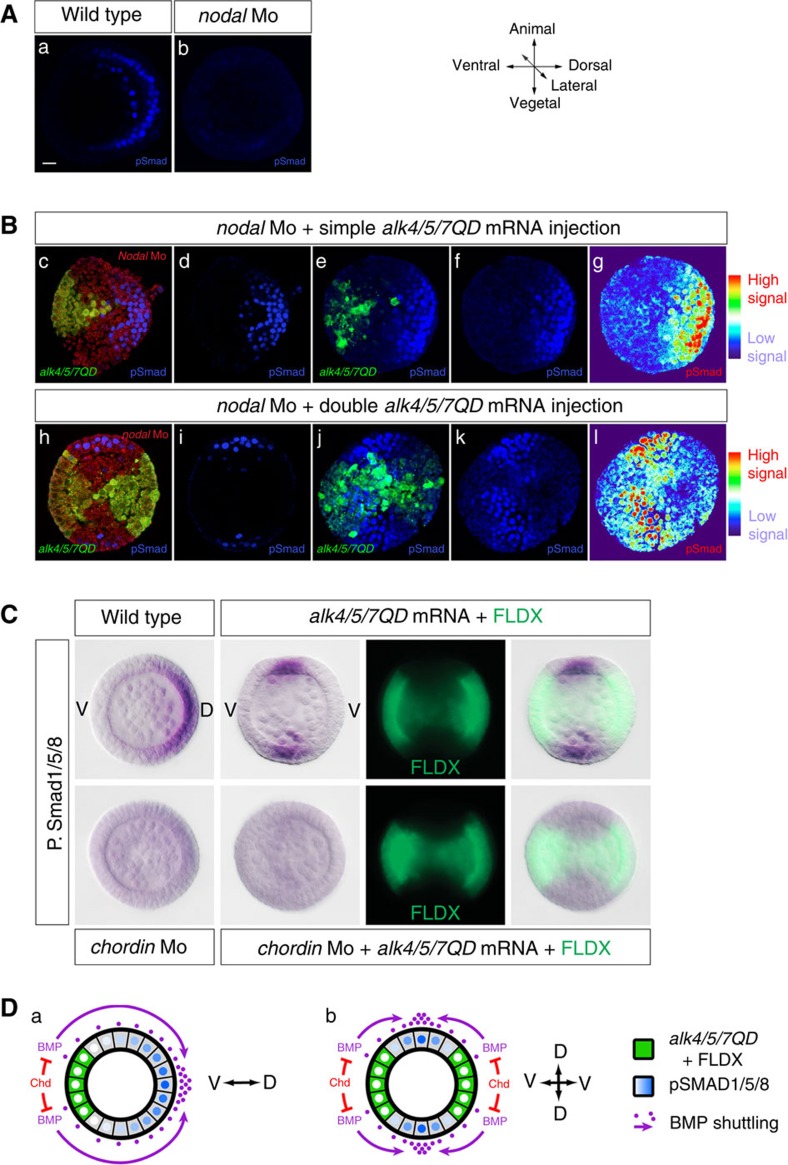
The pattern of Phospho-Smad1/5/8 in embryos with diametrically opposing sources of BMP2/4 provides evidence for shuttling of BMP2/4. (**A**) Phospho-Smad1/5/8 immunostaining at the mesenchyme blastula stage of control embryos (a) or *nodal* morphants (b). Strong nuclear staining is observed in a D/V gradient in one half of the control embryo, both in the ectoderm and in skeletogenic mesenchyme cells (a). Most of the signal disappears in the *nodal* morphants (b). (**B**) Phospho-Smad1/5/8 immunostaining of *nodal* morphants rescued by single injection of *alk4/5/7QD* mRNA at the 4-cell stage (c–g) and *nodal* morphants rescued by double injection of *alk4/5/7QD* mRNA (h-l). (c,h). The *nodal* morpholino was injected together with RLDX and the *alk4/5/7QD* mRNA with FLDX. Both tracers can be seen in the fully rescued embryo in (c). Single injection of *alk4/5/7QD* mRNA rescues psmad1/5/8 staining in *nodal* morphants on the opposite side of the injection clone (c–g) while double injection of *alk4/5/7* mRNA results in nuclear pSmad1/5/8 staining in two groups of cells located in lateral regions (h–l). Note that, unlike rescued embryos displaying high pSmad 1/5/8 signalling opposite to the ventral side (b,c), the highest intensity of pSmad1/5/8 staining is found in the midline separating the two organizers. Embryos in c, d, h and i are confocal stacks acquired with the exact same parameters. Embryos in e–g and j–l are maximum projections of confocal stacks acquired with the exact same parameters. Embryos in g and l are the same as in f and I but using the Fiji ‘Thermal' lookup table. (**C**) Accumulation of pSmad1/5/8 in the lateral regions of the doubly injected embryos requires Chordin function. Chordin morphants doubly injected with *alk4/5/7QD* do not show the strong bilateral pSmad staining but show instead a uniform and low staining. (**D**) Scheme summarizing the pattern of pSmad1/5/8 of wild type embryos (a) or of embryos doubly injected with Alk4/5/7QD (**b**). Both local repression of BMP signalling ventrally by Chordin and active shuttling of BMP2/4 from the ventral side to the dorsal side shape the steep gradient of pSmad1/5/8. Note that this configuration is partially similar to that of a *Drosophila* embryo in which Chordin produced in two ventrolateral territories shuttles Dpp to the dorsal midline. Scale bar, 10 μm.

In addition to duplicated skeletons, ventral sides and ciliary bands, two dorsal apex, where pairs of convergent and elongated spicules joined, were also recognizable in these double axis embryos. However, intriguingly, instead of originating from the same ventral side, the two spicules of each apex originated from two different ventral regions (Fig. 1C,D). The presence of two territories expressing *hox7* in gastrula stage embryos and of two dorsal apex in 72 h larvae were unexpected since in unperturbed embryos, the dorsal region is normally specified opposite to the ventral region. We therefore expected instead that misexpression of the activated Nodal receptor into two opposite blastomeres would inhibit specification of dorsal fates. To better understand these phenotypes, we tried to visualize BMP signalling at mesenchyme blastula stage (Fig. 3). In control embryos as well as in embryos rescued by a single injection of *alk4/5/7QD* mRNA, a gradient of phospho-Smad1/5/8 was detected on the dorsal side, opposite to the ventral clone of injection (Fig. 3A,B (a–d)). Embryos misexpressing *alk4/5/7QD* showed a striking pattern of phospho-Smad1/5/8 staining (Fig. 3Bh–l). In most (95% *n*>100) embryos doubly injected, strong phospho-Smad1/5/8 was detected at the centre of the two lateral regions that flanked the two ventral organizers. This pattern of phospho-Smad1/5/8 staining is consistent with BMP2/4 being shuttled towards the midline between the two Nodal expressing regions^13^(Fig. 3D). Consistent with the requirement for chordin in the process of shuttling, knocking-down chordin in these doubly injected embryos largely eliminated the strong asymmetrical pSmad1/5/8 signals normally detected at mesenchyme blastula, leaving only a weak and uniform residual pSmad1/5/8 staining (Fig. 3C). These data suggest that not only Chordin is required to block BMP signalling on the ventral side^30^ but it is also required for translocation of BMP2/4 to the dorsal side by acting as a shuttle as described in *Drosophila*^38^, *Xenopus*^13^, and *Nematostella*^39^.

### Two admp genes in the genomes of Echinoderms and Hemichordates

**Figure 4:**
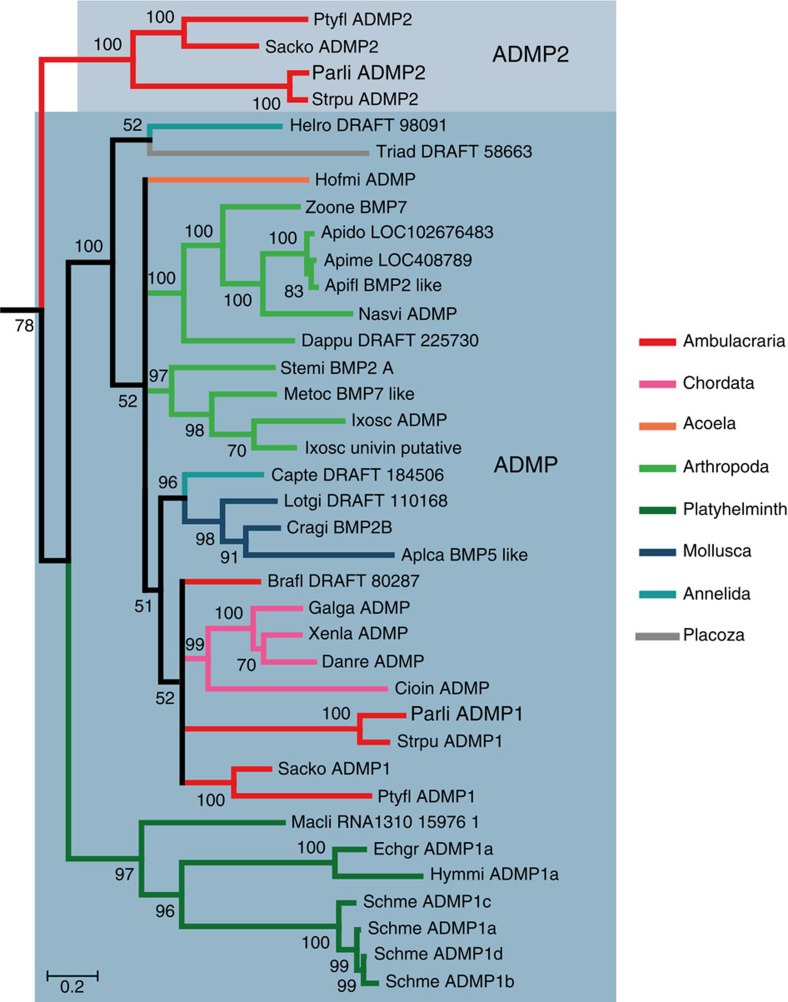
Phylogenetic analysis of sea urchin and various metazoan TGFβ ligands reveals *ADMP* gene duplication in Ambulacrarians. Bayesian analysis of ADMP ligands. Numbers above nodes correspond to posterior probabilities above 50%. Each branch of the tree is coloured according to the major phyla to which it belongs. Scale bar unit for branch length is the number of substitutions per site. Abbreviations for species name are the following. **Ambulacrarian species**: Parli: *Paracentrotus lividus*; Ptyfl: *Ptychodera flava*; Sacko: *Saccoglossus kowalevskii*; Strpu: *Strongylocentrotus purpuratus*. **Annelid species**: Capte: *Capitella teleta*; Helro: *Helobdella robusta*. **Arachnid species**: Ixosc: *Ixodes scapularis*; Metoc: *Metaseiulus occidentalis*; Stemi: *Stegodyphus mimosarum*. **Cephalochordate specie**: Brafl: *Branchiostoma floridae*. **Crustacean specie**: Dappu: *Daphnia pulex*. **Insect species**: Apido: *Apis dorsata*; Apifl: *Apis florea*; Apime: *Apis mellifera*; Nasvi: *Nasonia vitripennis*; Zoone: *Zootermopsis nevadensis*. **Mollusc species**: Aplca: *Aplysia californica*; Cragi: *Crassostrea gigas*; Lotgi: *Lottia gigantea*. **Placozoan specie**: Triad: *Trichoplax adhaerens*. **Platyhelminth species**: Echgr: *Echinococcus granulosus*; Hymmi: *Hymenolepis microstoma*; Macli: *Macrostomum lignano*; Schme: *Schmidtea mediterranea*. **Tunicate specie**: Cioin: *Ciona intestinalis*. **Vertebrate species**: Danre: *Danio rerio*; Galga: *Gallus gallus*; Xenla: *Xenopus laevis*. **Acoel specie**: Hofmi: *Hofstenia miamia*. This subtree was extracted from a phylogenetic tree including 192 TGFβ ligands provided in Supplementary Fig. 2. The complete list of accession numbers is provided in the supplementary methods.

Studies in *Xenopus* and zebrafish have identified the gene *admp* as an organizer specific gene and as a central component of D/V patterning during early development, allowing self-regulation and scaling on perturbations of BMP signalling^9^,^10^,^11^,^12^,^13^. The sea urchin genome contains two related *admp* genes named *admp1* and *admp2* (ref. 16). Interestingly, a pair of paralogous *admp* genes is also present in the genomes of the hemichordate *Saccoglossus kowaleskii* and *Ptychodera flava*. A Bayesian phylogenetic analysis using 192 TGF-β sequences sampled across the phylogenetic tree indicated that while sea urchin *admp1* grouped together with all the *admp* genes from deuterostomes and protostomes, sea urchin *admp2* was mostly related to *admp2* from *Saccoglossus* and *Ptychodera* and these orthologous *admp2* genes formed together a distinct branch within the family of *admp* genes (Fig. 4 and Supplementary Fig.1). This suggests that the duplication event that generated these paralogous genes likely preceded the separation of echinoderms and hemichordates.

### *admp1* and *admp2* are expressed at opposite poles of the D/V axis

**Figure 5:**
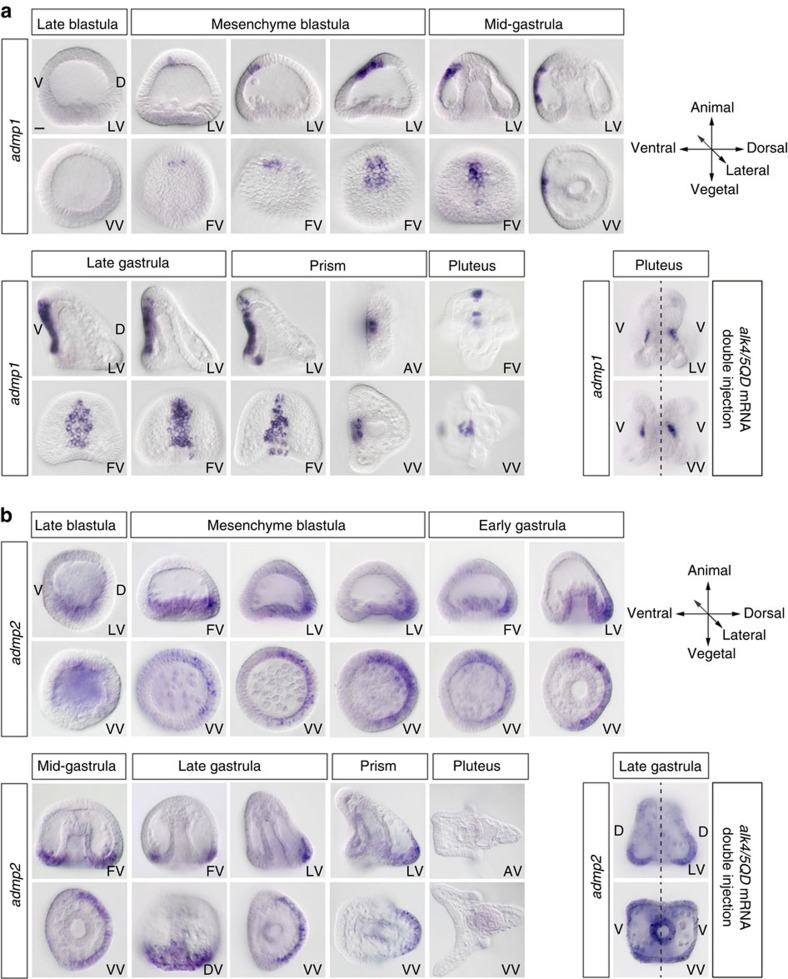
*admp1* is expressed in the ventral organizer while *admp2* is expressed in the dorsal-vegetal ectoderm. (**a**) *admp1* expression starts at mesenchyme blastula, first in a few ventral ectodermal cells near the animal pole, then it spreads vegetally to occupy a territory on the midline of the ventral ectoderm. At prism stage, novel territories expressing *admp1* appear in the animal pole region and in the ventral-most endoderm. In embryos with duplicated D/V axes, *admp1* is expressed at the centre of each oral lobe. (**b**) *admp2* expression is initiated at the late blastula stage in the dorsal-vegetal plate. During gastrulation *admp2* expression persists in a thin layer of dorsal-vegetal ectodermal cells and at prism stage in cells that will give rise to the dorsal apex of the pluteus larva. In embryos with duplicated D/V axes, *admp2* is expressed in two opposing territories corresponding to the duplicated dorsal-vegetal ectoderm. Scale bar, 10 μm.

*admp1* expression was first detected at mesenchyme blastula stage in a small cluster of three to six contiguous cells near the animal pole region (Fig. 5a). During gastrulation, the size of the *admp1* expression territory extended from the animal pole towards the vegetal region to occupy a roughly rectangular region comprising about 20–30 cells on the ventral midline of the gastrula. At late gastrula and prism stages, *admp1* expression occupied a six-cell wide belt of cells crossing the whole ventral ectoderm in the midline. Finally at late prism stage, one additional cluster of elongated neural-like cells in the animal pole region and another group of cells at the junction between the endoderm and ectoderm on the midline, started to express *admp1*. Strikingly, in larvae with duplicated D/V axes, *admp1* was expressed within each duplicated oral lobe in two discrete regions in the middle of each oral lobe revealing the high level of patterning of these Nodal induced territories. In conclusion, this analysis revealed that *admp1* is expressed in an intriguing pattern in the ventral midline of the gastrula that expresses *nodal* and that has organizing activity.

In contrast, *admp2* expression started at the onset of gastrulation in the vegetal most presumptive ectoderm on the dorsal side (Fig. 5b). During gastrulation, expression of *admp2* remained restricted to a thin layer of ectodermal cells immediately overlying the PMCs on the dorsal side and at late gastrula and prism stages, it occupied the dorsal lip of the blastopore and the dorsal-vegetal apex. This analysis revealed that, although *admp1* and *admp2* are the products of a relatively recent gene duplication event and although they both encode BMP-like ligands, their expression patterns have diverged radically so that they are now expressed at opposite poles along the D/V axis. These non-overlapping expression patterns suggest that these two genes have also functionally diverged.

### Opposite regulation of *admp1* and *admp2* by Nodal and BMP signalling

**Figure 6:**
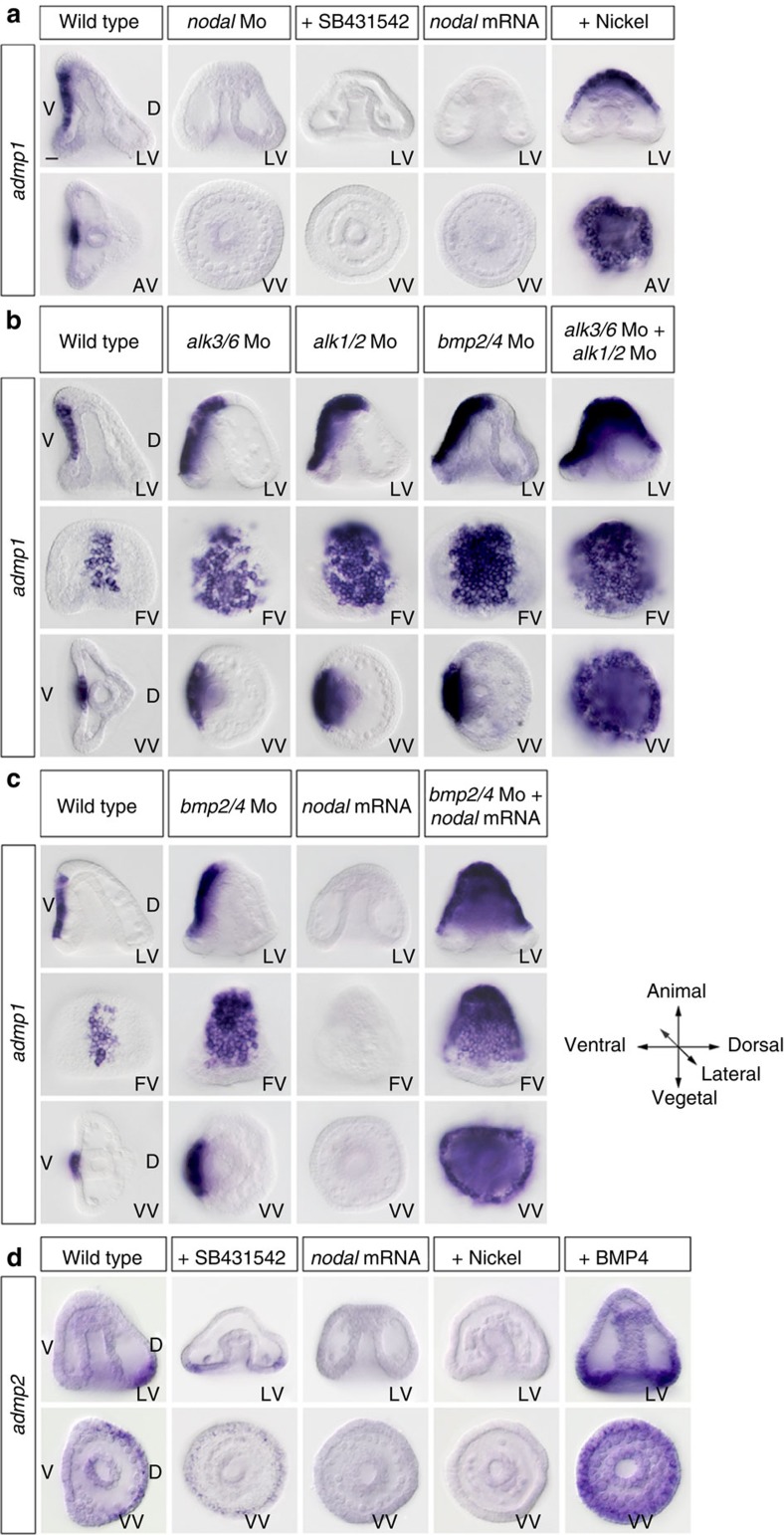
Opposite regulation of *admp1* and *admp2* by BMP signalling. (**a**) *admp1* expression is abolished in *nodal* morphants and following inhibition of Nodal signalling by SB431542. *admp1* expression is also suppressed by overexpression of *nodal* but it is strongly upregulated in nickel treated embryos. (**b**) Expression of *admp1* at gastrula stage in control embryos and following knockdown of various components of the BMP signalling pathway as indicated. Blocking BMP signalling using either a morpholino targeting *bmp2/4*, or the BMP type I receptor *alk3/6*, or the BMP type I receptor *alk1/2* causes a partial extension of *admp1* while blocking translation of both *alk3/6* and *alk1/2* transcripts with a combination of morpholinos causes a massive extension of *admp1* expression to most of the ectoderm. (**c**) A massive derepression of *admp1* is also observed after combining inhibition of *bmp2/4* mRNA translation and overexpression of *nodal*. (**d**) Expression of *admp2* is expanded ventrally following treatment with recombinant BMP4 and suppressed by overexpression of *nodal* or treatment with nickel. Expression of *admp2* persists in a thin layer of dorsal-vegetal cells following inhibition of Nodal signalling by treatment with SB431542. LV, lateral view; VV, vegetal view; FV, frontal view, AV, animal view. Scale bar, 10 μm.

As predicted, from its expression in the ventral ectoderm, transcription of *admp1* was abolished by injection of a morpholino targeting the *nodal* transcript or by treatment with SB431542, a potent and specific inhibitor of the Nodal receptor (Fig. 6a). However, unexpectedly, overexpression of *nodal* (Fig. 6a) or treatment with recombinant Nodal protein (Supplementary Fig. 2B) did not upregulate but eliminated *admp1* expression. Intriguingly, treatment with the ventralizing agent nickel chloride strongly upregulated *admp1* expression and caused massive ectopic expression throughout the ectoderm. That nickel treatment and Nodal overexpression have opposite effects on expression of *admp1* suggests that while both treatments expand ventral fates, they may do so by different mechanisms.

In contrast, consistent with its dorsal expression, *admp2* expression was expanded ventrally at mesenchyme blastula stage following treatment with recombinant BMP4 and it was eliminated following overexpression of *nodal* (Fig. 6d). Interestingly, *admp2* expression persisted in the circum-blastoporal ectoderm following inhibition of Nodal signalling with SB431542 treatment^37^. This suggests that *admp2* expression is induced in part by signals produced in the dorsal-vegetal region that are independent of Nodal, as well as by BMP signals.

### Transcriptional repression of *admp1* is conserved in the sea urchin organizer

Studies in *Xenopus* and zebrafish demonstrated that *admp* expression is repressed by BMP signalling and that this property allows *admp* to function as a sensor of BMP signalling^7^. Treatment with as little as 0.1 μg ml^−1^ of BMP4, a concentration that does not cause dorsalization, eliminated *admp1* expression suggesting that the negative regulation of *admp1* expression by BMP signalling observed in vertebrates is conserved in the sea urchin (Fig. 6b,c and Supplementary Figure 2A). Injection of a morpholino targeting either the *bmp2/4* or *alk3/6* (ref. 30) or *alk1/2* (ref. 22) transcripts dramatically upregulated *admp1* expression, which expanded both laterally and towards the animal pole to occupy about half of the ventral region(Fig. 6b) while in the *alk3/6*;*alk1/2* double morphants, *admp1* was massively ectopically expressed in most of the ectoderm. Taken together, these results demonstrate that the negative regulation of *admp* by BMP signalling that has been described in the organizer of vertebrates is conserved in the D/V organizer of the sea urchin. We were however, intrigued by the fact that nickel treatment, like inhibition of both BMP type I receptors induces a massive ectopic expression of *admp1*, while overexpression of *nodal* results in suppression of *admp1* expression. We therefore reasoned that since *bmp2/4* is a transcriptional target of Nodal signalling, one possible explanation for the repression of *admp1* by Nodal overexpression was that *nodal* overexpression induced BMP2/4 expression, which in turn repressed *admp1* transcription. To test this hypothesis we overexpressed *nodal* in *bmp2/4* morphants (Fig. 6c). Indeed, the combination of *nodal* overexpression and knock down of *bmp2/4* caused a massive ectopic expression of *admp1* similar to that observed following inhibition of the two BMP type I receptors or following treatment with nickel (Fig. 6a,c). The effect of nickel on *admp1* is highly similar to the effect of blocking translation of both BMP type I receptors, and is even more potent than blocking the function of BMP2/4 itself, the major regulator of BMP signalling in the sea urchin embryo. This observation suggests one possible mechanism for the enigmatic action of nickel on sea urchin embryos. It suggests that nickel exerts its ventralizing action by inhibiting BMP signalling.

### ADMP1 and ADMP2 promote pSmad1/5/8 signalling

**Figure 7:**
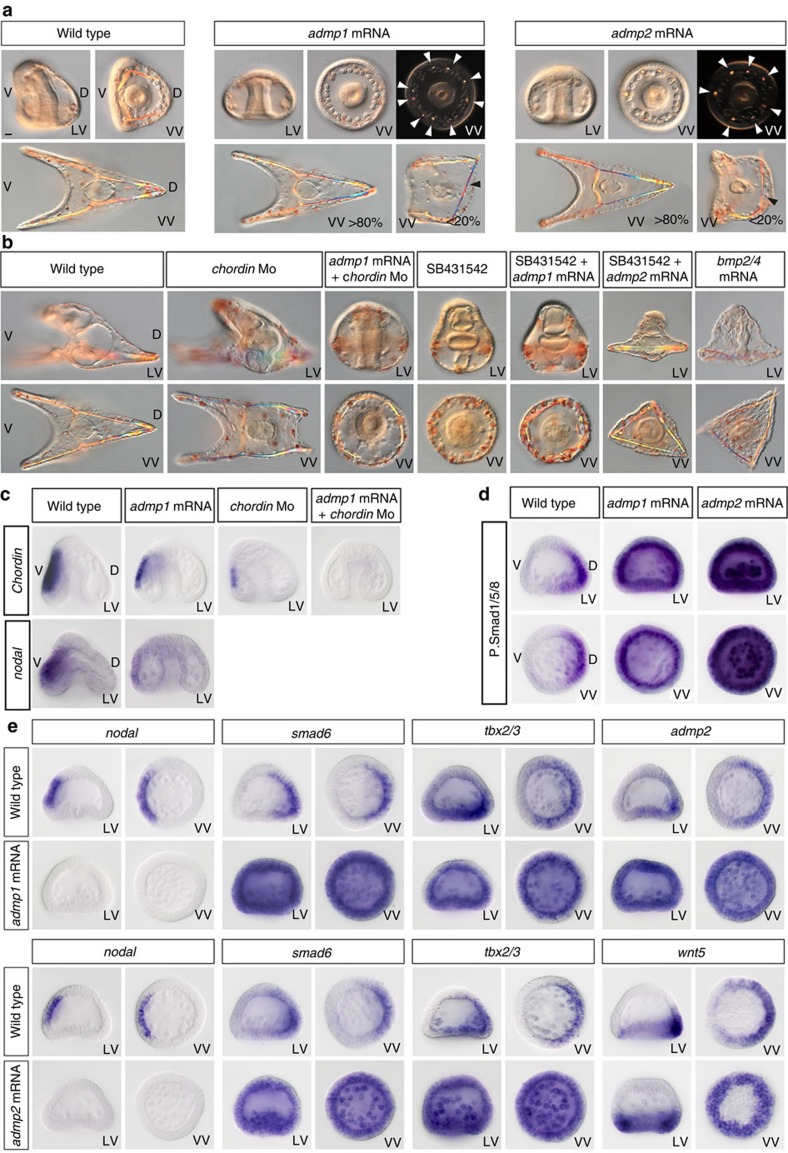
*admp1* and *admp2* act as prototypical BMP ligands but display divergent activities in specific contexts. (**a**) Morphological phenotypes resulting from overexpression of *admp1* and *admp2*. Overexpression of *admp1* or *admp2* at 1,000 μg ml^−1^ completely radialized the embryos at gastrula stage. Note the presence of multiple spicule rudiments and the axial position of the gut in *admp1* and *admp2* overexpressing gastrulae. However, at pluteus stage, most embryos overexpressing *admp1* or *admp2* recovered a largely normal D/V axes and developed into normal pluteus. A fraction of these larvae displayed minor skeletal defects such as ectopic spicules elements. (**b**) Overexpression of *admp2* suppressed formation of the ciliary band of SB431542 treated embryos and promoted growth and elongation of the skeleton resulting in a phenotype similar to that caused by overexpression of *bmp2/4 or of bmp5/8*. Overexpression of *admp1* but not of *admp2* in a *chordin* morpholino background caused 100% of the embryos to develop with a Nodal loss-of-function phenotype. (**c**) *chordin* expression is reduced in embryos overexpressing *admp1* or in *chordin* morphants and eliminated in *chordin* morphants overexpressing *admp1*. (**d**) Both ADMP1 and ADMP2 promote pSmad1/5/8 signalling. pSmad1/5/8 antibody staining in control embryos and in embryos injected with *admp1* or *admp2* mRNAs. While the pSmad1/5/8 signal is restricted to the dorsal side of control embryos at mesenchyme blastula stage, overexpression of *admp1* or of *admp2* induces massive and strong phosphorylation of Smad1/5/8 in the whole ectoderm. In addition, overexpression of *admp2*, but not of *admp1*, induces strong pSmad1/5/8 in the primary mesenchymal cells. (**e**) Molecular analysis of *admp1* or *admp2* overexpressing embryos. At mesenchyme blastula stage, overexpression of *admp1* or *admp2* suppresses the expression of *nodal* and expands the expression of the dorsal marker genes *smad6* and *tbx2/3*.*admp1* overexpression also expands *admp2* while overexpression of *admp2* expands *wnt5*. Scale bar, 10 μm.

We then tested if ADMP1 and ADMP2 act as prototypical BMP ligands and if, like BMP2/4, they promote specification of dorsal territories when overexpressed. During gastrulation, embryos overexpressing *admp1* or *admp2* appeared completely radialised as evidenced by the radial arrangement of the PMCs and the presence of multiple spicule rudiments around the archenteron (Fig. 7a). Consistent with this idea, overexpression of either *admp1* or *admp2* induced massive and strong activation of pSmad1/5/8 signalling throughout the ectoderm (Fig. 7d and Supplementary Fig. 3). Interestingly, overexpression of *admp2*, but not of *admp1*, also induced strong pSmad1/5/8 signalling in the PMCs, suggesting that ADMP2 has a specific function in promoting pSmad1/5/8 signalling in the skeletogenic mesoderm, an activity consistent with its expression in the dorsal-vegetal ectoderm overlying the PMCs. At mesenchyme blastula stage, embryos overexpressing *admp1* or *admp2* failed to express *nodal* (Fig. 7e) and instead ectopically expressed the dorsal marker genes *smad6* and *tbx2/3*, consistent with the ubiquitous activation of pSmad1/5/8 signalling caused by overexpression of either *admp1* or *admp2* (Fig. 7e). Furthermore, *admp2* expression was expanded ventrally in embryos overexpressing *admp1*, indicating that ADMP1 signalling can promote *admp2* expression, and *wnt5* was expanded ventrally in embryos overexpressing *admp2*. At early gastrula stage, a partial recovery of the expression of *nodal* and *chordin* was observed in *admp1* and *admp2* overexpressing embryos but the expression of *FGFA* and *pax2/5/8* was abolished while the expression of the dorsal markers *msx*, *tbx2/3* and *wnt5* was expanded ventrally (Supplementary Fig 4). Strikingly, despite the strong and ubiquitous activation of pSmad1/5/8 signalling and the dramatic changes in gene expression observed at mesenchyme blastula and early gastrula, the vast majority of embryos overexpressing *admp1* or *admp2* developed into pluteus larvae with a relatively normal D/V axis but carrying moderate defects at the level of the skeleton (Fig. 7a). We never observed any dorsalized embryos following overexpression of *admp1* or *admp2* suggesting either that the activity of ADMP1 and ADMP2 is significantly weaker than that of BMP2/4 or that ADMP1 and ADMP2 are unstable proteins that are rapidly degraded. In comparison, all the embryos injected with *bmp2/4* mRNA at 1,000 μg ml^−1^ were completely and irreversibly dorsalized^27^ (Fig. 7b). One hypothesis that would explain why *admp1* or *admp2* overexpression has only transient and relatively modest effects on D/V patterning is that Chordin may buffer the effect of *admp1* or *admp2* overexpression. To test this hypothesis, we overexpressed *admp1* or *admp2* in *chordin* morphants (Fig. 7b). Indeed, while injection of *admp1* alone only transiently perturbed D/V patterning, in the absence of Chordin, *admp1* caused 100% of the embryos to develop with a typical Nodal loss of function phenotype. Thus, in the absence of Chordin, overexpression of *admp1*, abolishes *nodal* expression, mimicking the effects of overexpression of low doses of *bmp2/4* (ref. 22) or *bmp5/8* (Supplementary Fig. 5), consistent with previous reports indicating that in *Xenopus*, ADMP competes with Nodal for binding to ACVRII^40^. Also, consistent with its weak dorsalizing activity and/or stability, overexpression of *admp1* failed to repress formation of the ciliary band in embryos deprived of Nodal signalling. In contrast, overexpression of *admp2* had dramatic effects on spiculogenesis and patterning of the ectoderm of embryos devoid of Nodal signalling. While the ectoderm of embryos treated with SB431542 developed into a prominent ciliary band covering a disorganized and poorly differentiated skeleton, the ectoderm of embryos treated with SB431542 but that overexpressed *admp2* developed into a thin dorsal-like epithelium. Furthermore, these embryos contained elongated spicules in the vegetal region, a phenotype reminiscent of the phenotype of embryos overexpressing an activated BMP receptor^30^ or *bmp2/4* (Fig. 7b). We conclude that although ADMP1 and ADMP2 both encode closely related BMP ligands, specific activities of each one of these factors can be revealed in particular contexts. On overexpression, ADMP1 but not ADMP2 is able to antagonise Nodal signalling in the absence of Chordin. In contrast, ADMP2 but not ADMP1 is able to suppress formation of the ciliary band, to dorsalize the ectoderm and to rescue patterning and growth of the spicules in the absence of Nodal signalling.

### ADMP1 and ADMP2 cooperate with BMP2/4 to build the dorsal apex

**Figure 8:**
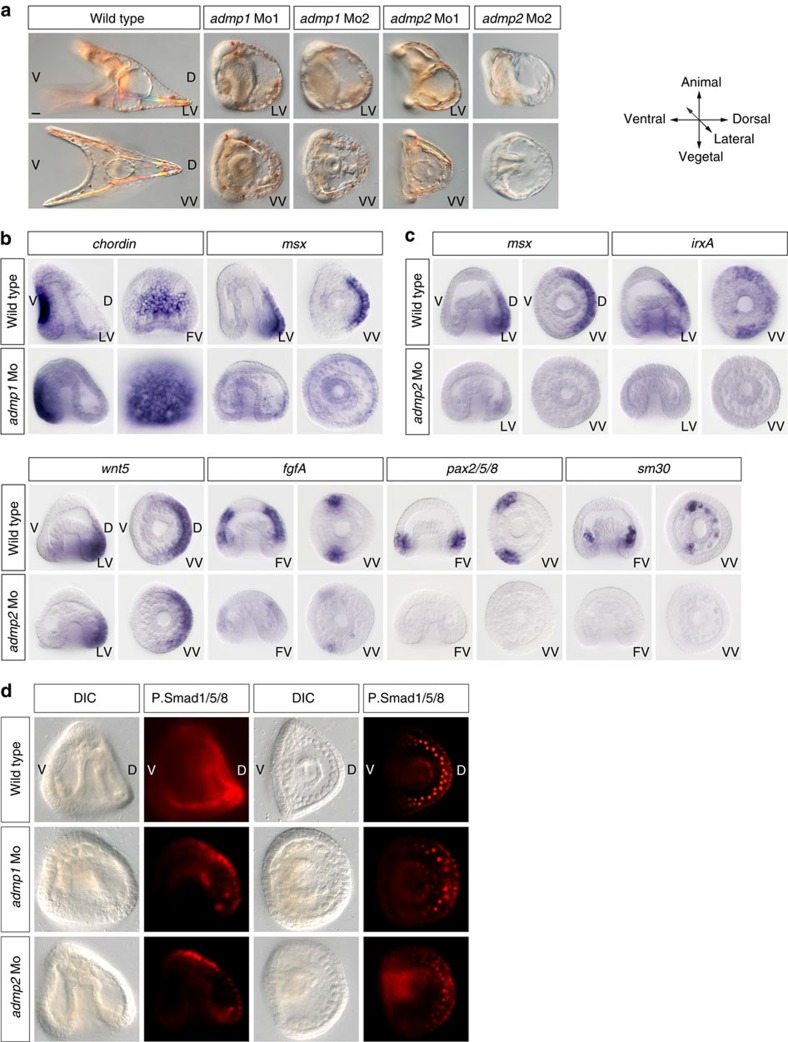
ADMP1 and ADMP2 are required for high level of BMP signalling and cooperate with BMP2/4 to build the dorsal apex. (**a**) Morphological phenotypes caused by knocking-down ADMP1 or ADMP2 with morpholino oligonucleotides. Injection of morpholinos targeting the translation start site (Mo1) or the 5' UTR (Mo2) of *admp1* or *admp2* disrupts D/V patterning resulting in embryos that do not elongate along the ventral or dorsal sides. (**b**) Knocking-down *admp1* with *admp1-Mo2* increases the size of the *chordin* expressing territory consistent with the idea that ADMP1 antagonizes Nodal. The dorsal marker gene *msx* is also downregulated in *admp1* morphants. (**c**) Knocking-down *admp2* with *admp2-Mo1* specifically eliminated the expression of marker genes expressed in the dorsal-vegetal and lateral ectoderm including *fgfA*, *pax2/5/8*, *irxA* and *msx* and suppressed expression of *sm30* in the PMC clusters. (**d**)Morpholinos targeting either *admp1* or *admp2* strongly reduced pSmad1/5/8 staining in the presumptive dorsal-vegetal ectoderm and mesoderm but knocking-down *admp2* with *admp2-Mo1* more strongly diminished pSmad1/5/8 staining in the PMCs compared with knocking-down *admp1* with *admp1-Mo2.* Scale bar, 10 μm.

**Figure 9:**
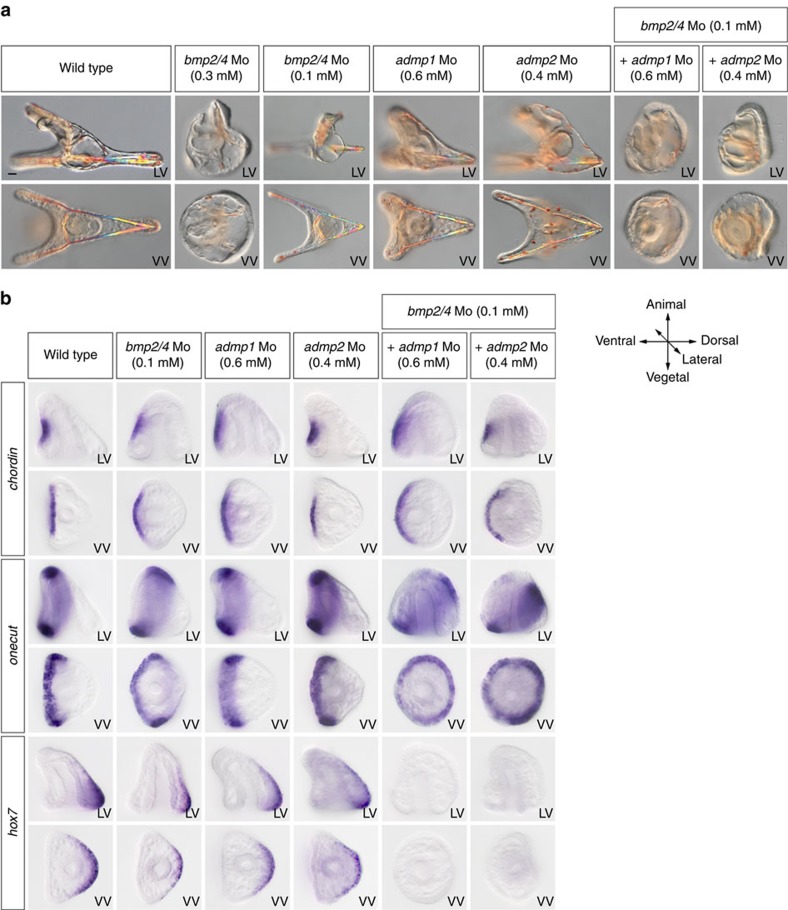
ADMP1 and ADMP2 act in synergy with BMP2/4 to repress formation of the ciliary band on the dorsal side. (**a**) Blocking translation of BMP2/4 causes a dramatic expansion of the ciliary band on the dorsal side while partially inhibiting BMP2/4 causes a truncation of the dorsal apex. Co-injection of low doses of the *bmp2/4* together with low, sub-optimal, doses of the *admp1* or *admp2* morpholinos produces a strong BMP loss-of-function phenotype indicating that these two ADMP ligands cooperate with BMP2/4 during D/V axis formation. Note the similarity of the phenotypes of embryos injected with high doses of the BMP2/4 morpholino or co-injected with the *admp1*+*bmp2/4* or *admp2*+*bmp2/4* morpholinos at sub-optimal doses. (**b**) Expression of *chordin*, *onecut* and *hox7* in embryos co-injected with low doses of the *bmp2/4* and *admp1* or *bmp2/4* and *admp2* morpholinos. Note the dramatic dorsal expansion of the ciliary band marker *onecut*, the loss of *hox7* and the partial expansion of *chordin* following co-injection of either combination of the two morpholinos at low doses. Scale bar, 10 μm.

To test whether *admp1* and *admp2* are required for D/V patterning, we designed two different non-overlapping antisense morpholinos oligonucleotides against each transcript. Injection of *admp1* morpholinos resulted in embryos that failed to elongate along the D/V axes and that displayed signs of increased Nodal signalling such as expansion of the spatial expression domain of *chordin* (Fig. 8a) as well as signs of decreased BMP signalling such as reduced pSmad signalling in the dorsal-vegetal ectoderm at gastrula stage and reduced expression of the dorsal marker gene *msx* (Fig. 8b,d). Consistent with the expression of *admp2* in the lateral and vegetal most ectoderm, knocking-down *admp2* produced embryos with a reduced dorsal apex (Fig. 8a) and abolished the expression of marker genes of these territories such as *irxA*, *msx*, *fgfA* and *pax2/5/8* as well as of the marker of PMC clusters *sm30* (Fig. 8c). However, the *admp2* morphants had a normal expression of *wnt5* suggesting that *admp2* may act in parallel or downstream of *wnt5*. Also, knocking-down *admp2* strongly reduced pSmad1/5/8 signalling in the dorsal string of PMCs at gastrula stage, consistent with the idea that ADMP2 is required for patterning of the PMCs on the dorsal side (Fig. 8d). To further test if ADMP1 and ADMP2 cooperate with BMP2/4 and appreciate the relative contributions of each factor to D/V patterning, we performed a synergy assay (Fig. 9). We co-injected embryos with low, sub-optimal doses of the *admp1* or *admp2* morpholino, doses which do not cause any morphological phenotype, together with low doses of the *bmp2/4* morpholino that result in truncation of the dorsal apex without expansion of the ciliary band. While each injection alone resulted in either no phenotype (*admp1-Mo*, *admp2-Mo*) or a moderate phenotype (*bmp2/4-Mo*), co-injection of low doses of *admp1+bmp2/4* or of *admp2+bmp2/4* morpholinos produced embryos that were rounded, devoid of pigment cells and that displayed a prominent ciliary band covering the whole dorsal region (Fig. 9a). Expression of the dorsal marker *hox7* was suppressed in these embryos and the ciliary band marker *hnf6* was ectopically expressed in the presumptive dorsal ectoderm territory (Fig. 9b). This phenotype, which is similar to that caused by a failure of BMP2/4 signalling^30^, strongly suggests that both ADMP1 and ADMP2 act in synergy and cooperate with BMP2/4 to specify the dorsal and dorsal-vegetal territories during D/V patterning.

## Discussion

Although manipulations leading to axis duplications have been instrumental in identifying the Spemann organizer and in analysing D/V patterning in vertebrates^5^,^41^, experimental manipulations consistently leading to full duplications of the D/V axes had never been described outside chordates and whether D/V organizers are present in non-chordates embryos was not known. Hörstadius and Lindahl occasionally observed sea urchin larvae with duplicated D/V axes following fusion of embryos or stretching of eggs into a thin pipette^42^,^43^. However, besides these exceptional cases, there was until now no consistent protocol allowing the efficient generation of embryos with duplicated D/V axes. We have reported here that ectopic expression in *nodal* morphants of the activated receptor for the TGF beta Nodal into two opposite blastomeres at the four-cell stage efficiently induced duplication of the D/V axes in single embryos, resulting in larvae with two sets of ventral, dorsal and ciliary band structures. These findings confirm the cardinal role of Nodal as a master gene regulating morphogenesis of the sea urchin embryo^31^,^44^. Embryos with two sources of Nodal develop with two skeletons since Nodal controls the spatial expression of FGFA and VEGF, which in turn will guide migration of the primary mesenchyme cells that will build the skeleton^45^,^46^. Nodal also controls specification of the ventral ectoderm and, through expression of BMP2/4, of the dorsal ectoderm that together with the vegetal ectoderm will build the dorsal apex^30^. Indeed, embryos with two sources of Nodal formed two fully patterned oral lobes and two dorsal apex, although there was a considerable reorganization of pattern regarding the location of the presumptive dorsal regions in these larvae.

The finding that embryos with two sources of Nodal formed two precisely shaped ciliary bands running at the junction between the ventral and dorsal territories was unexpected and is particularly striking. It is consistent with the idea that Nodal and BMP specify the ectoderm and restrict the position of the ciliary band at the interface of the ventral and dorsal territories. It is also consistent with the idea that formation of the ciliary band requires Chordin and Lefty being produced downstream of Nodal signalling and locally inhibiting Nodal and/or BMP2/4 signalling at the interface of the ventral and dorsal regions and creating an environment compatible with ciliary band formation as previously proposed^23^,^37^,^47^. However, that Nodal signalling cell autonomously induced neural markers in the vegetal ciliary band is also highly suggestive of a more direct role of Nodal in induction of the ciliary band and therefore of induction of neural fates, at least in the vegetal part of the ciliary band. Understanding how Nodal and BMP together with Lefty and Chordin so precisely chisel the position and width of the ciliary band will be a challenge in the future^48^.

The striking organizing activity of Nodal in the sea urchin is undoubtedly due to the fact that Nodal acts upstream of both BMP2/4 and Chordin in this organism. Ectopic expression of Nodal is therefore sufficient to define both the ventral and dorsal sides of an embryo as well as to induce tissues that normally form at the interface between them. Indeed, it has been shown recently that creating two opposing gradients of Nodal and BMP4 at the animal pole of the zebrafish blastula is sufficient to organize a fully patterned embryonic axis^49^.

The Siamese larvae also illustrate the renowned developmental plasticity and regulative properties of the sea urchin embryo. Following generation of two Nodal expressing signalling centres, the sea urchin embryo is capable of reorganizing the whole D/V pattern of its ectoderm and mesoderm to generate a pluteus larvae, containing two ventral sides, two dorsal sides and two ciliary bands from a gastrula with an apparently normal number of cells. Formation of these well-proportioned ectopic structures raises an intriguing question: what is the mechanism that allows the adjustment of pattern with size in these Siamese larvae? The expansion–repression mechanism, in which production of a diffusible factor that positively regulates a morphogen gradient, is repressed by morphogen signalling, has been proposed to control this process of scaling^50^,^51^,^52^. Indeed, BMP2/4 and Nodal form together a prototypical expansion—repression mechanism since Nodal promotes BMP2/4 expression whereas BMP signalling antagonizes Nodal signalling^22^,^30^,^31^. Therefore, an increase in the intensity of Nodal signalling will cause an increase in *bmp2/4* expression that in turn will attenuate Nodal signalling and *nodal* expression. It is therefore likely that this feedback mechanism of BMP on Nodal signalling, which plays a central role in D/V patterning during normal development, also plays a key role in scaling pattern with size in these twinned embryos.

A marked difference between echinoderms and vertebrates regarding the BMP-ADMP circuit is that in vertebrates, ADMP and BMP2/4 are expressed in opposing territories are under opposite transcriptional control. This is not the case in the sea urchin embryo since both *bmp2/4* and *admp1* are regulated by Nodal signalling. This raises an intriguing question: which BMP ligand amplifies the ADMP1 signal when it reaches the dorsal side, if *bmp2/4* and *admp1* are not expressed dorsally? Indeed, *admp2* is the perfect candidate for this function. First, *admp2* is expressed with the same kinetics as *admp1*, starting at the onset of gastrulation. Second, *admp2* is expressed on the presumptive dorsal side, that is, opposite to the *admp1* expression territory. Finally, *admp2*, unlike *admp1*, is positively regulated by BMP signalling^37^. Indeed, we have shown that overexpression of *admp1* strongly upregulates *admp2* expression. Therefore, *admp1* and *admp2* are expressed at opposite poles and are under opposite transcriptional controls as are *admp1* and *bmp4* in vertebrates. Any decrease in BMP2/4 signalling will cause *admp1* expression to increase and following translocation to the dorsal side, ADMP1 protein will upregulate the expression of *admp2* dorsally. Conversely, an increase in BMP signalling on the dorsal side will antagonize *nodal* expression on the ventral side leading to a decrease of BMP2/4 and ADMP1 production and secondarily to a decrease of a*dmp2* expression.

In this study, we uncovered an essential role for *admp2* in specification of the dorsal-vegetal and lateral-vegetal ectoderm. The lateral-vegetal ectoderm that overlies the PMC clusters plays a central role in positioning the clusters of PMCs and in promoting growth and patterning of the spicules. However, how this lateral ectoderm is specified is not completely understood. By analysing the consequences of perturbing Nodal and BMP signalling on *fgfA* and *pax2/5/8*, the first described markers of the border ectoderm, Rottinger *et al*. and Saudemont *et al*. first demonstrated that Nodal and BMP2/4 signalling act to position rather than to induce the lateral-vegetal ectoderm^37^,^46^ (Röttinger *et al*. Fig. 2), a finding later confirmed by McIntyre *et al*. and by the identification of Wnt5 as one of the signals required for specifying the lateral-vegetal ectoderm^53^. Similarly, it has been proposed that the dorsal-vegetal ectoderm, which overlies the dorsal ring of PMCs, is specified by mechanisms that are in part independent of Nodal and BMP2/4 but that are dependent on Wnt signalling (see Fig. 11f,g in Saudemont *et al*.^37^). In this study, we have shown that *admp2* is expressed in the dorsal-vegetal ectoderm and that ADMP2 is required for specification of the lateral-vegetal ectoderm. Furthermore, we found that ADMP2 signals preferentially to the PMCs and is essential for elongation of the dorsal spicules and of the apex. These findings emphasize the essential role played by BMP signalling in specification of the vegetal ectoderm and in morphogenesis of the skeleton. In summary, these data suggest that although *admp1* and *admp2* are likely the products of a gene duplication in the ambulacraria lineage, in the sea urchin embryo, these genes are expressed at opposite poles of the D/V axis, are subject to opposite regulations by Nodal and BMP2/4 signalling and they display signalling specificities in different germ layers. Nevertheless, both genes act in concert and play key roles in the D/V gene regulatory network: *admp1* acts as a sensor of BMP signalling and is a central component of a self-regulatory circuit that autoregulates the level of BMP signalling, while *admp2* has adopted a novel and essential role in formation and patterning of the vegetal and lateral ectoderm that controls growth and patterning of the dorsal part of the skeleton.

**Figure 10:**
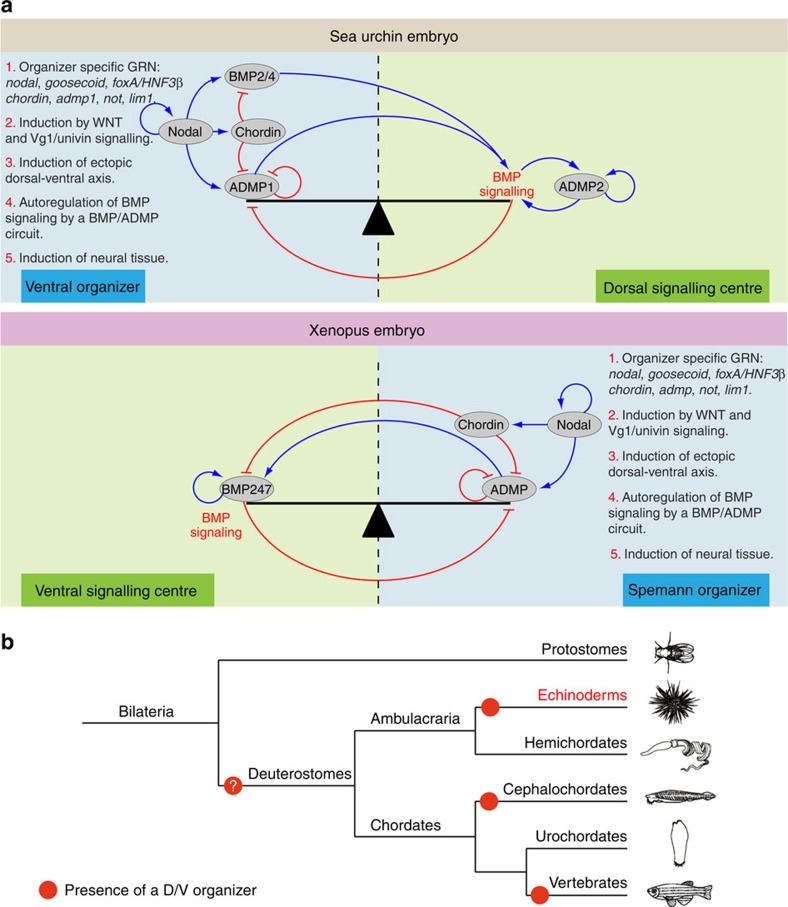
Homologous gene regulatory networks drive formation of the D/V organizer in chordates and in the sea urchin. (**a**) Topology of the BMP-ADMP-Chordin network in chordates and echinoderms. In chordates, BMP2/4/7 ligands are expressed in a ventral signalling centre while *admp* is expressed on the opposite side. BMP signalling on the ventral side promotes expression of BMP ligands and represses *admp* expression, (repression). Nodal signalling in the Spemann organizer promotes expression of ADMP^8^,^10^,^11^ and Chordin^70^. Chordin then shuttles BMP and ADMP ligands towards the ventral side where they activate BMP signalling (expansion)^7^,^13^. Chordin inhibits ADMP signalling dorsally. In the sea urchin, Nodal also positively regulates the expression of *chordin* and of an organizer specific BMP ligand, *admp1*, but Nodal also controls the expression of *bmp2/4*. Chordin inhibits ADMP and BMP2/4 signalling ventrally and promotes translocation of these ligands to the opposite dorsal side where they activate BMP signalling (expansion). BMP signalling on the dorsal side does not activate *bmp2/4* expression but induces *admp2*, which can in turn autoregulate. BMP signalling on the side opposite to the organizer represses expression of *admp1* in the organizer by an unknown mechanism (repression). A historical continuity of the D/V organizer of chordates and echinoderms is suggested by the striking conservation at the level of the whole gene regulatory network (GRN) which includes key transcription factors and signalling molecules such as *nodal*, *lefty*, *bmp2/4*, *chordin*, *admp*, *not*, *lim1* and *HN3β*/*foxA.* Both GRNs require Wnt and Univin/Vg1 signalling to start and the activity of both GRNs can endow cells with organizer-like properties and coherently induce a whole set of tissues along the D/V axis. Finally, both organizers are involved in induction of neural tissue mainly through BMP inhibition and also possibly through direct induction by Nodal in the case of the sea urchin embryo. (**b**) Phylogeny of deuterostomes and phyla where a D/V organizer was characterized functionally. The red dot indicates the presence of a D/V organizer. A D/V organizer is present in all chordates, with perhaps the exception of tunicates. A D/V organizer is also present in echinoderms, suggesting that the evolutionary origin of the organizer may be traced back, at least, to the common ancestor of deuterostomes.

That ectopic Nodal expression generates larvae with duplicated D/V axes and that the BMP ADMP-Chordin circuit operates downstream of Nodal in the sea urchin adds more weight to our previous proposal that the ventral ectoderm of the sea urchin embryo is a D/V organizing centre that shares similarities with the Spemann organizer^27^,^31^,^37^,^54^,^55^. Several lines of evidence point to a common evolutionary origin of the sea urchin D/V organizer and the Spemann organizer of Chordates (Fig. 10). First, the sea urchin D/V organizer like the Spemann organizer, requires Wnt and Vg1/Univin signalling to form and is induced by Nodal signalling^5^,^24^,^27^,^56^,^57^. Second, the idea that in chordates, the Spemann organizer works along the D/V axes primarily as a source of BMP inhibitors that will antagonize the activity of BMP ligands and induce neural tissues applies to echinoderms as well since in the sea urchin Chordin, the main inhibitor of BMP signalling in this organism^30^,^58^, is produced in the ventral organizer and ectopic Nodal signalling induces formation of an ectopic ciliary band at the border of the *nodal* expressing territory. A third argument in favour of this hypothesis is that, in addition to Chordin, many components of the gene regulatory network that defines the Spemann organizer including *goosecoid*, *HNF3β*/*foxA*, *not*, *lim1*, *brachyury* as well as *nodal*, *lefty*, *chordin* and *admp* are also components of the gene regulatory network that drives D/V patterning in the sea urchin^59^,^60^. A fourth argument that supports the homology between the sea urchin D/V organizer and the Spemann organizer is the conserved role of ADMP as an organizer specific gene negatively regulated by BMP signalling. Finally and importantly, we have shown here that following induction of an ectopic source of Nodal, a full complement of D/V structures are induced consistent with the idea that Nodal expressing cells are indeed a large-scale and long-range organizing centre functionally analogous to the Spemann organizer of chordates. Taken together, these findings suggest that the evolutionary origin of the Spemann organizer may be more ancient than previously thought and suggest that this origin may be traced back to the common ancestor of deuterostomes.

How far can we trace back the evolutionary origin of the D/V organizer? The idea of an even more ancient evolutionary origin of a D/V organizer is strongly suggested by molecular and embryological studies on secondary axis formation in different clades including ecdysozoa, lophotrocozoa and cnidarians^18^,^19^,^20^,^39^,^61^,^62^,^63^. Interestingly, the ability to regulate and to give rise to well-proportioned embryos after bisection is not restricted to deuterostomes but is also observed in insects^64^. Furthermore, although ADMP, a central component of the BMP autoregulatory system is absent from *Drosophila*, *Tribolium* and from nematods, orthologs of this gene are encoded in the genomes of insect species, crustacean and chelicerates^65^,^66^ (Fig. 4) and both *nodal* and *admp* are present in the genomes of molluscs and annelids^17^,^67^. It will therefore be very interesting to investigate the function of Nodal and to determine if *admp* and genes encoding Dpp-like ligands are under opposite transcriptional control in these organisms.

Finally, studies in Nematostella showed that establishment of a secondary axis of polarity relies on Dpp signalling and on shuttling by Chordin strongly suggesting that bilateral symmetry evolved before the split of bilateria and cnidaria^39^,^62^,^68^ and raising the intriguing possibility that the evolutionary origin of the D/V organizer may be traced further back to the basis of the phylogenetic tree.

## Methods

### Animals, embryos and treatments

Adult sea urchins (*Paracentrotus lividus*) were collected in the bay of Villefranche-sur-Mer. Embryos were cultured at 18 °C in Millipore-filtered sea water and at a density of 5,000 per ml. Fertilization envelopes were removed by adding 1 mM 3-amino-1,2,4 triazole 1 min before insemination to prevent hardening of this envelope followed by filtration through a 75-μm nylon net.

Treatments with SB431542 were performed by adding the chemical diluted from stocks in dimethylsulphoxide in 24-well plates protected from light at the desired time. As controls, dimethylsulphoxide was added alone at 0.1% final concentration. Treatments by these inhibitors were performed continuously starting after fertilization. Treatments with recombinant Nodal or BMP4 protein (R&D SYSTEMS; 0.1–0.5 μg ml^−1^) were started at the 16-cell stage. Treatments with NiCl_2_ were performed by exposing embryos to 0.3 mM of chemical stating 30 min after fertilization.

### Cloning of the *admp1* and *alk1*/2 cDNAs

A full-length *admp1* and *alk1/2* complementary DNAs were identified from a collection of *P.lividus* ESTs (http://octopus.obs-vlfr.fr/). The complete sequence of these clones was determined. The Genebank accession numbers of *admp1*, *admp2* and *alk1/2* are respectively: KP968256, KT276376 and KF498643.

### Phylogenetic analysis

Sequences were retrieved from various databases using blast search or keyword search and aligned using Clustal Omega (www.clustal.org/omega/) with default parameters. Alignment was manually checked for obvious errors using Aliview (www.ormbunkar.se/aliview/) then trimmed using Trimalv1.3 (trimal.cgenomics.org/) with user defined parameters (Min. percentage position to conserve: 18, Gap threshold: 0.7, Similarity threshold: 0, Window size: 1). Bayesian phylogenetic analysis was done using MrBayes 3.2.5, mrbayes.sourceforge.net/) with a mixed amino-acid substitution model, and 5 millions generations. Consensus tree was generated after discarding 25% generations as burn-in.

### Overexpression analysis and Morpholino injections

For overexpression studies, the coding sequence of the genes analysed was amplified by PCR with the Pfx DNA polymerase (Invitrogen) using oligonucleotides containing restriction sites and cloned into pCS2. Capped mRNAs were synthesized from NotI-linearized templates using mMessage mMachine kit (Ambion). After synthesis, capped RNAs were purified on Sephadex G50 columns and quantitated by spectrophotometry. RNAs were mixed with Rhodamine Dextran (10,000 MW) or Fluoresceinated Dextran (70,000 MW) at 5 mg ml^−1^ and injected in the concentration range 100–1,200 μg ml^−1^. For simple axis rescue and double axis induction experiments, *nodal* mRNAs were injected at 500 μg ml^−1^ and *alk4/5/7 Q265D* was injected at 800 μg ml^−1^ . *admp1* and *admp2* mRNAs were injected at 500–1,200 μg ml^−1^ . *bmp2/4* and *nodal* mRNAs were injected at 400 μg ml^−1^ . Morpholino antisense oligonucleotides were obtained from Gene Tools LLC (Eugene, OR). Characterization of the *nodal*, *BMP2/4*, *Chordin and Alk3/6* morpholinos has been described in^30^,^54^. Since morpholinos can have side effects, display toxicity or produce variable reductions in gene activity, we designed and tested two different morpholinos against *admp1* and *admp2* and we verified that both produced similar results. We also injected as control a morpholino targeting the hatching enzyme, which does not perturb patterning of the embryo and allows to control for the developmental delay caused by the injection. The phenotypes observed were consistent with the zygotic expression pattern of the gene tested and with previous well-established functional data. The sequences of all the morpholino oligomers used in this study are listed below (the nucleotides corresponding to the initiator codon ATG are underlined).

*hatching enzyme-Mo:5*′- GCAATATCAAGCCAGAATTCGCCAT-3′

*admp1-Mo1-ATG*: 5′-ACACGAAAATAATCTCCATTGTCTT-3′

*admp1-Mo2-UTR*: 5′-TAGAAAGCCGCAATCGAAACACAGT

*alk1/2-Mo-ATG*: 5′-TAAATTCTAGTCGTCGCGTCGCCAT -3′

*admp2-Mo1-ATG*: 5′- TAGGGCAAAATTAGGCATCATCATG-3′

*admp2-Mo2-UTR*: 5′- TCGATTTCGTCCGCCTTCCAGCATC-3′

Approximately 2 pl of solution were injected. All the injections were repeated multiple times and for each experiment >100 embryos were analysed. Only representative phenotypes present in at least 80% of the injected embryos are presented. To control for non-specific defects caused injections of the morpholino, a morpholino targeting the hatching enzyme transcript was injected as a control in the initial testing of each morpholino. Similarly to control for non-specific defects caused by mRNA overexpression, a control mRNA encoding either beta galactosidase or a zebrafish Pitx2 transcript containing a frame shift were used as controls.

### In situ hybridizations

The *nodal*, *chordin*, *foxA*, *fgfA*, *pax2/5/8*, *tbx2/3*, *msx*, *irxA*, *hox7*, *wnt5*, *foxG*, *onecut*, *gata1/2/3* and *gcm*, have been described previously^31^,^37^,^44^,^46^,^69^. The *admp1* and *admp2* probes were derived from full-length complementary DNAs cloned in pSport. Probes derived from pBluescript vectors were synthesized with T7 RNA polymerase after linearization of the plasmids by NotI, while probes derived from pSport were synthesized with SP6 RNA polymerase after linearization with XmaI. Control and experimental embryos were developed for the same time in the same experiments. Fluorescent *in situ* hybridization was performed using a florescein coupled *onecut* probe and an anti-fluorescein antibody coupled to an Alexa fluorophore. Three-dimensionalreconstructions of stained embryos was done using Fiji's ‘3D Viewer' plug-in (http://fiji.sc/Fiji). Detection of the lineage tracer was performed using an anti-fluorescein antibody coupled to alkaline phosphatase and using Fastred as substrate.

### Anti-phospho-Smad1/5/8 immunostaining

The antibody we used is an anti-phospho-Smad1/5/8 from Cell Signalling (Ref 9511) raised against a synthetic phosphopeptide corresponding to residues surrounding Ser463/465 contained in the motif SSVS of human Smad5. Embryos were fixed in paraformaldehyde 4% in MFSW for 15 min then briefly permeabilized with methanol. Embryos were rinsed once with PBST, four times with PBST-BSA 2% and incubated overnight a +4 °C with the primary antibody diluted 1/400 in PBST supplemented with 2% BSA. Embryos were washed 6 times with PBST-BSA 2%, then the secondary antibody diluted in PBST-BSA 2% was added to the embryos. In all cases the antibody was incubated overnight at +4 °C. For immunofluorescence, the secondary antibody was washed six times with PBST. For Alkaline phosphatase revelation, two rinses were made with PBST following the secondary antibody incubation and two with TBST. Embryos were washed twice with the alkaline phosphatase buffer supplemented with Tween 0.1% and staining was performed with NBT and BCIP as substrates at the final concentration of 50 mM each. In both cases staining was stopped by four rinses with PBST+EDTA 5 mM then two rinses with PBST containing glycerol at 25 and 50%. Embryos were mounted in a drop of the Citifluor anti-bleaching mounting medium, then observed under a conventional fluorescence microscope or with a confocal microscope.

## Additional information

**Accession codes:** The *admp1*, *admp2* and *alk1/2* sequences generated in this study have been deposited in GenBank nucleotide database under accession codes KP968256, KT276376 and KF498643.

**How to cite this article:** Lapraz, F. *et al*. A deuterostome origin of the Spemann organizer suggested by Nodal and ADMPs functions in Echinoderms. *Nat. Commun.* 6:8434 doi: 10.1038/ncomms9434 (2015).

## Supplementary Material

Supplementary Figures and Supplementary Methods Supplementary Figures 1-5 and Supplementary Methods

Supplementary Movie 1 3D modelisation of a 72h Siamese larvae obtained by double injection of alk4/5/7QD mRNA injections in two opposite blastomeres at the 4-cell stage of nodal morphants

Supplementary Movie 2 3D modelling of a Siamese gastrula stained by fluorescent in situ hybridization with a onecut probe.

Supplementary Movie 3 3D modelling of a Siamese gastrula stained to label the progeny of the alk4/5/7QD injection clones.
