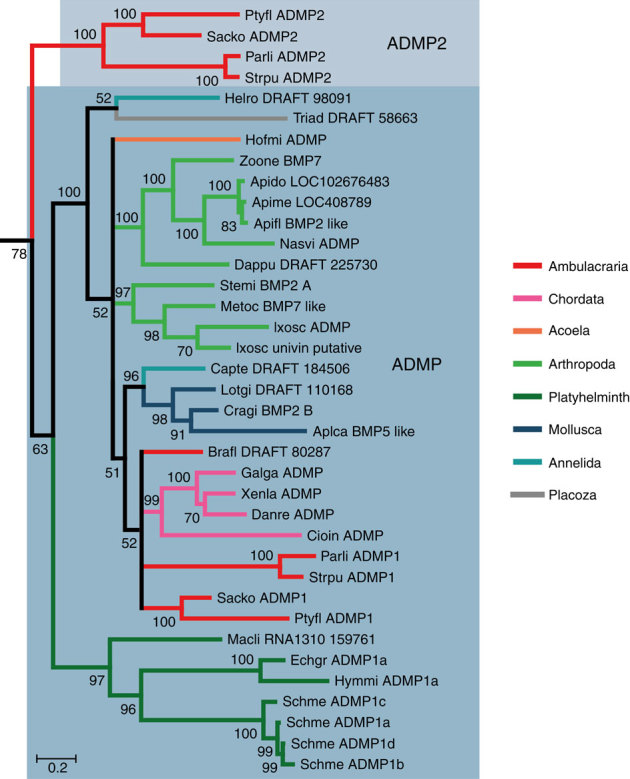# Erratum: A deuterostome origin of the Spemann organiser suggested by Nodal and ADMPs functions in Echinoderms

**DOI:** 10.1038/ncomms9927

**Published:** 2015-11-19

**Authors:** François Lapraz, Emmanuel Haillot, Thierry Lepage

**Keywords:** Developmental biology, Evolutionary developmental biology, Cell signalling

10.1038/ncomms9434

In Fig. 4 of this Article, the bootstrap value associated with the node that separates the two main ADMP clades was inadvertently omitted during the production process. The correct version of the figure appears below. Additionally, the funding information for this Article was not acknowledged. The Acknowledgements should have included:

This work was supported by the Centre National de la Recherche Scientifique (CNRS), a grant from the Agence Nationale de la Recherche (ANR) to T.L. (ANR-14-CE11-0006-01) and by support from the Association pour la Recherche sur le Cancer (ARC) (grant 7801 and SFI20121205586) to T.L., E.H. and F.L. were supported by grants from the Ministère de la Recherche et de l'Enseignement Supérieur and by a 4th year of PhD fellowship from the ARC.

**Figure 4**