# Supplementary material for: A deuterostome origin of the Spemann organiser suggested by Nodal and ADMPs functions in Echinoderms
Source: Nat Commun. 2015 Oct 1;6:8434. doi: 10.1038/ncomms9434 (PMC4600745; doi:10.1038/ncomms9434)
Supplement: Supplementary Figures and Supplementary Methods — Supplementary Figures 1-5 and Supplementary Methods [file ncomms9434-s1.pdf]

Supplementary Figure 1.

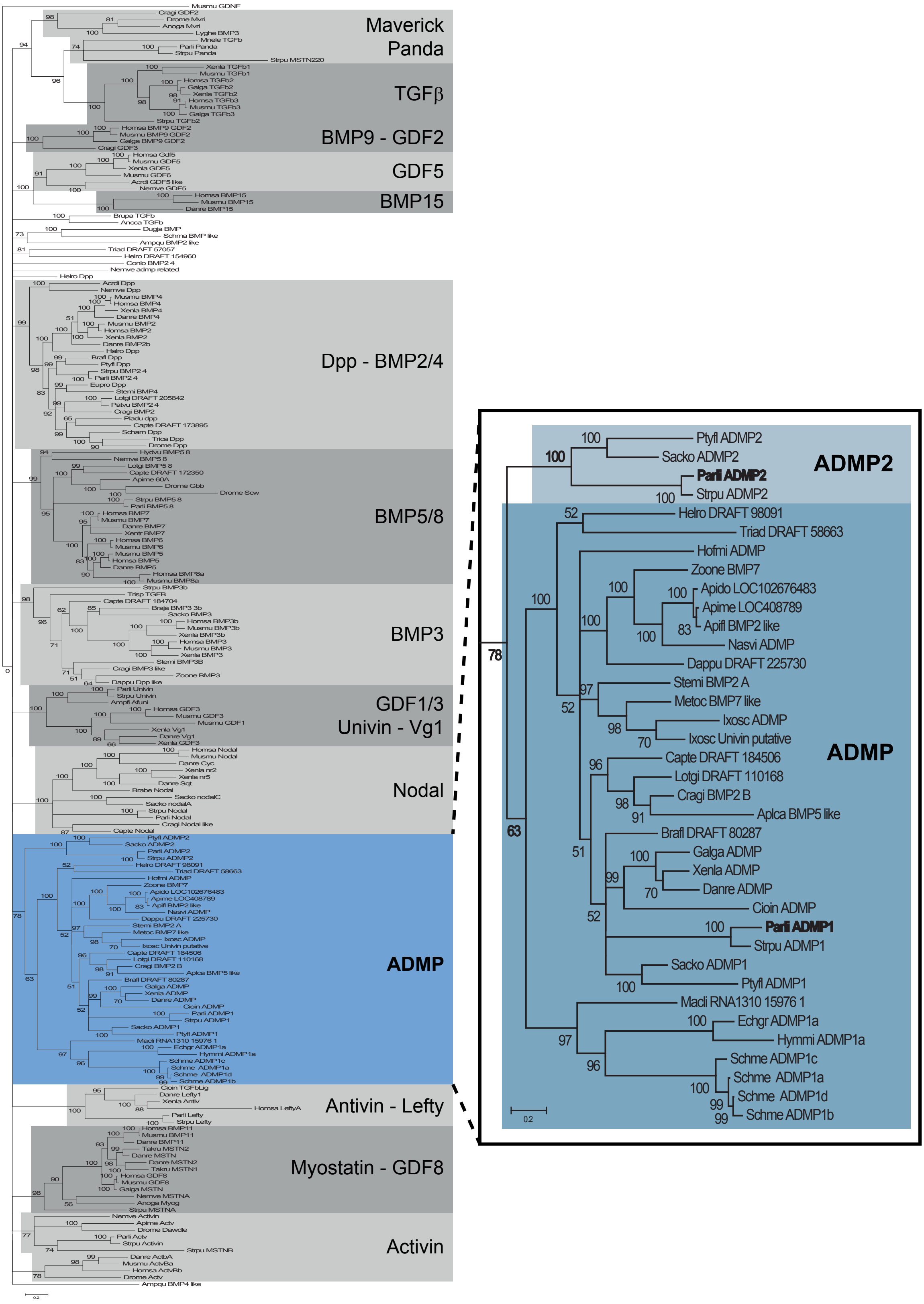

Phylogenetic analysis of sea urchin and various metazoan TGFβ ligands.
Bayesian phylogenetic tree including 192 various TGFβ ligands. Enlarged subtree corresponds to ADMP ligands. Numbers above nodes correspond to posterior probabilities above 50%. Scale bar unit for branch length is the number of substitutions per site. Musmu_GDNF sequence was used to root the tree. Abbreviations for species name are the following. **Ambulacrarian species**: Ampfi: *Amphiura filiformis*; Parli: *Paracentrotus lividus*; Ptyfl: *Ptychodera flava*; Sacko: *Saccoglossus kowalevskii*; Strpu: *Strongylocentrotus purpuratus*. **Annelid species**: Capte: *Capitella teleta*; Helro: *Helobdella robusta*; Pladu: *Platynereis dumerilii*. **Arachnid species**: Ixosc: *Ixodes scapularis*; Metoc: *Metaseiulus occidentalis*; Stemi: *Stegodyphus mimosarum*. **Cephalochordate species**: Brabe: *Branchiostoma belcheri*; Brafl: *Branchiostoma floridae*; Braja: *Branchiostoma japonicum*. **Cnidarian species**: Acrdi: *Acropora digitifera*; Acrmi: *Acropora millepora*; Nemve: *Nematostella vectensis*; Hydvu: *Hydra vulgaris*. **Crustacean specie**: Dappu: *Daphnia pulex*. **Ctenophoran specie**: Mnele: *Mnemiopsis leidyi*. **Insect species**: Anoga: *Anopheles gambiae*; Apido: *Apis dorsata*; Apifl: *Apis florea*; Apime: *Apis mellifera*; Drome: *Drosophila melanogaster*; Nasvi: *Nasonia vitripennis*; Scham: *Schistocerca americana*; Trica: *Tribolium castaneum*; Zoone: *Zootermopsis nevadensis*. **Mollusc species**: Aplca: *Aplysia californica*; Cragi: *Crassostrea gigas*; Lotgi: *Lottia gigantea*; Patvu: *Patella vulgata*. **Nematode species**: Ancca: *Ancylostoma caninum*; Brupa: *Brugia pahangi*; Trisp: *Trichinella spiralis*. **Onichophoran specie**: Eupro: *Euperipatoides rowelli*. **Placozoan specie**: Triad: *Trichoplax adhaerens*. **Platyhelminth species**: Dugja: *Dugesia japonica*; Echgr: *Echinococcus granulosus*; Hymmi: *Hymenolepis microstoma*; Macli: *Macrostomum lignano*; Schma: *Schistosoma mansoni*; Schme: *Schmidtea mediterranea*. **Poriferan specie**: Ampqu: *Amphimedon queenslandica*. **Tunicate species**: Cioin: *Ciona intestinalis*; Halro: *Halocynthia roretzi*. **Vertebrate species**: Danre: *Danio rerio*; Galga: *Gallus gallus*; Homsa: *Homo sapiens*; Musmu: *Mus musculus*; Takru: *Takifugu rubripes*; Xenla: *Xenopus laevis*; Xentr: *Xenopus tropicalis*. **Acoel species**: Conlo: *Convolutriloba longifissura*; Hofmi: *Hofstenia miamia*.
Overexpression of ADMP1 or ADMP2 induces pSmad signalling in embryos devoid of Nodal signalling.

Supplementary Figure 2.

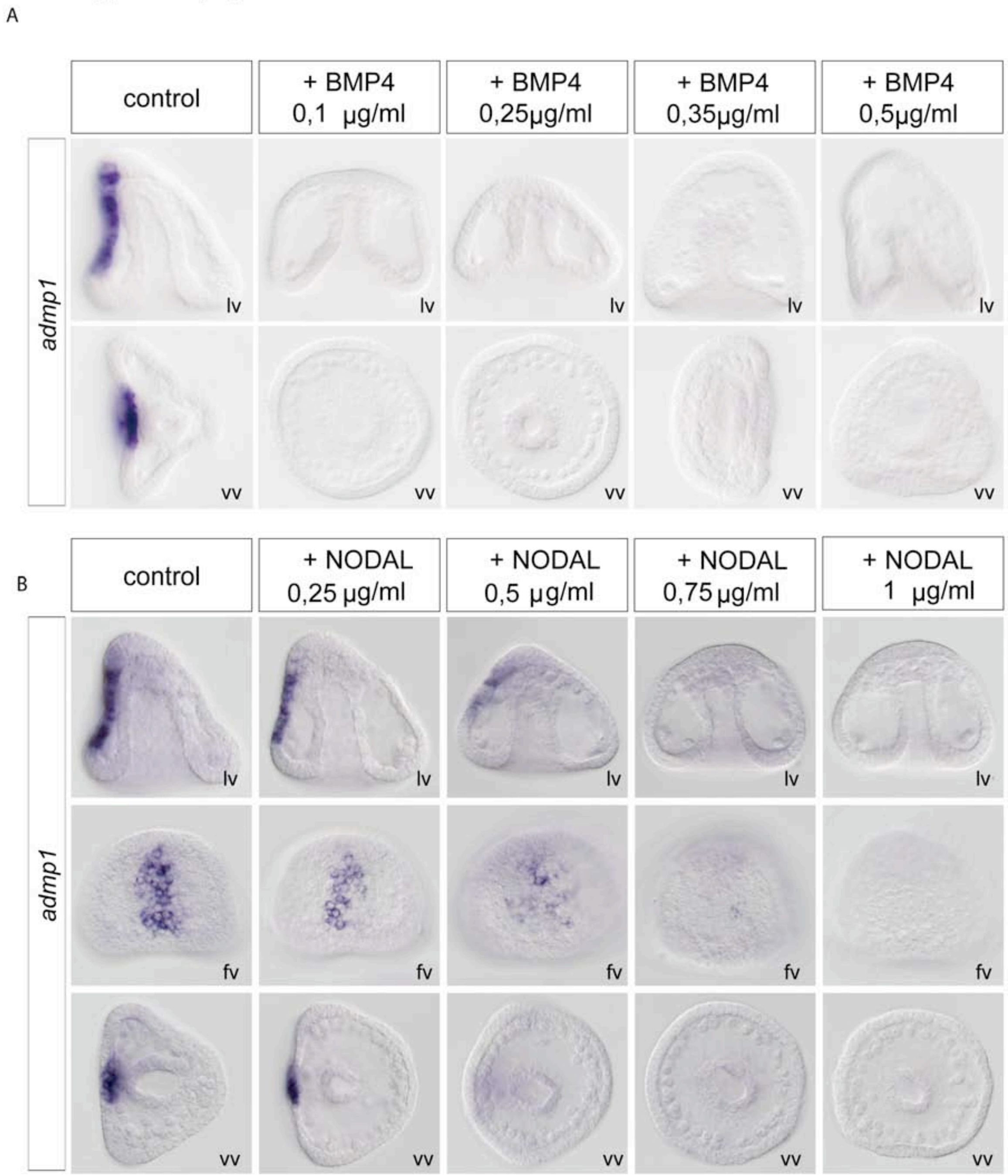

Effects of treatments with recombinant Nodal and BMP2/4 on *admp1* expression
(A) Inhibition of *admp1* expression by treatment with recombinant BMP4 protein. *admp1* expression was abolished following treatment with BMP4 at 0,1 µg/ml, a concentration that did not dorsalize sea urchin embryos.
(B) Effect of treatment with recombinant Nodal protein on *admp1* expression. Treatments with increasing concentrations of recombinant mouse Nodal protein progressively eliminated *admp1* expression and at 0.75 µg/ml, *admp1* expression was abolished.

Supplementary Figure 3.

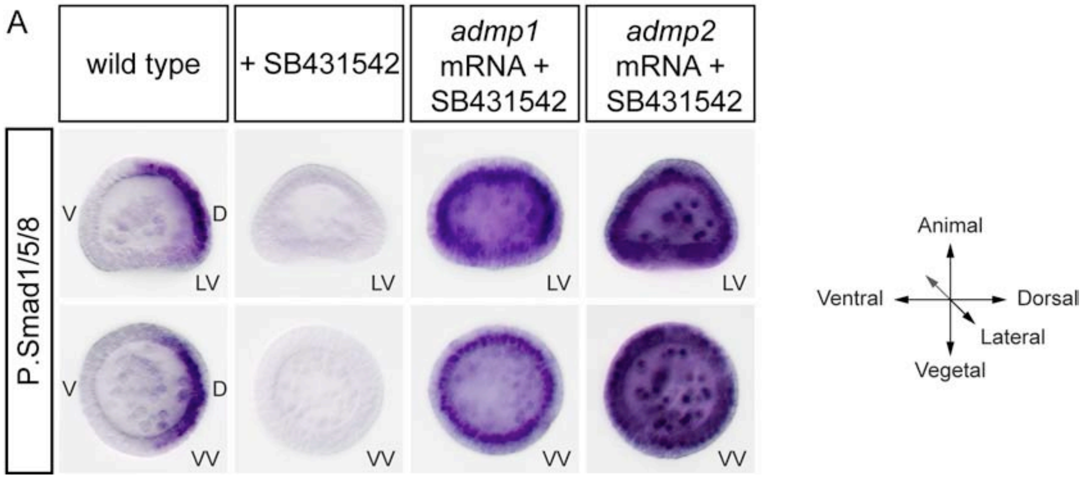

Overexpression of *admp1* or of *admp2* directly induces pSmad1/5/8 phosphorylation

Embryos overexpressing *admp1* or *admp2* were treated with SB431542, fixed at mesenchyme blastula and processed for pSmad1/5/8 immunostaining. Both ADMP1 and ADMP2 induced strong Smad1/5/8 phosphorylation in the presence of SB431542 suggesting that the strong activation of pSmad is caused by direct signalling of ADMP1 and ADMP2 rather than by induction of *bmp2/4*.

Supplementary Figure 4.

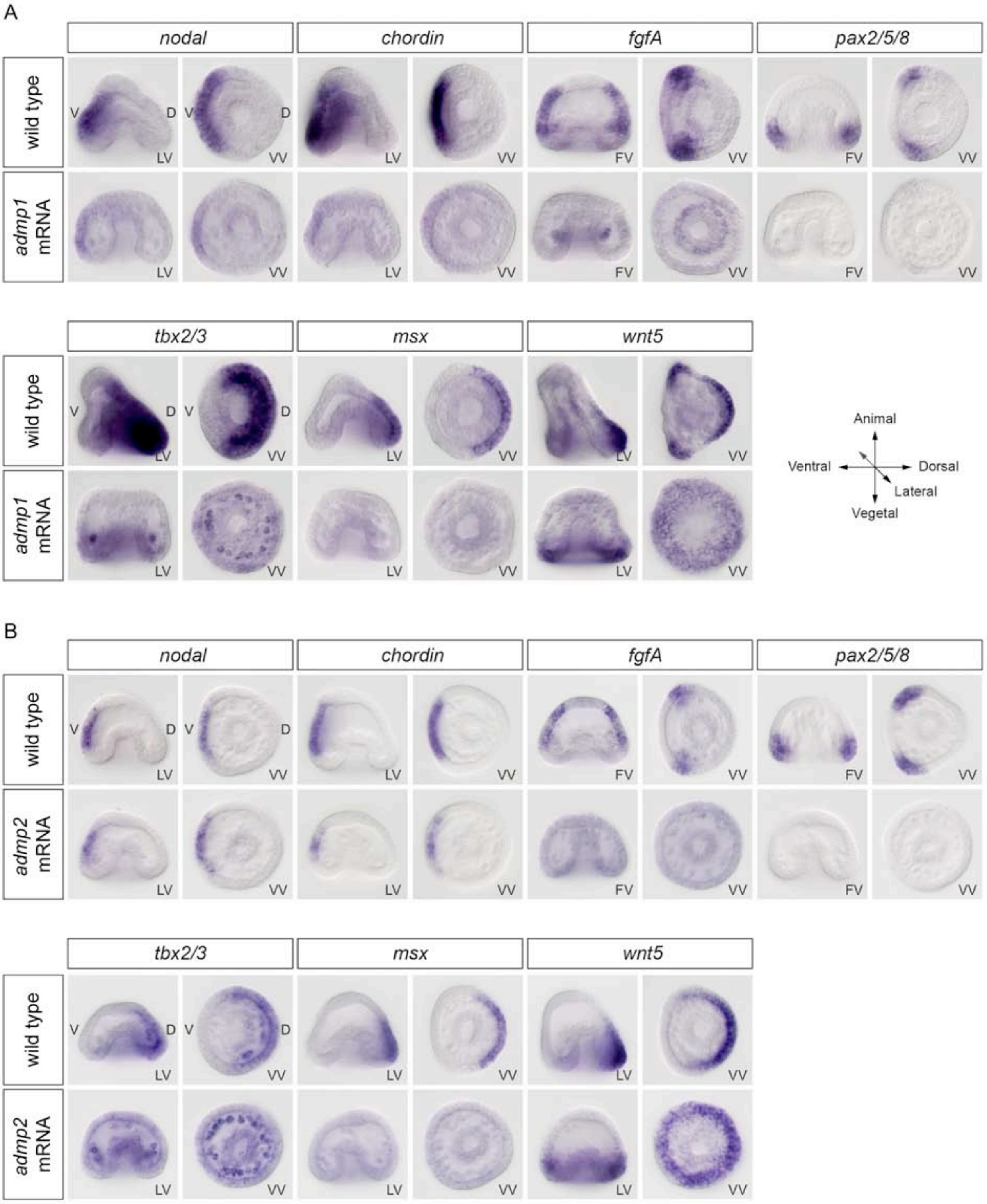

Expression of marker genes at gastrula stage following overexpression of *admp1* and *admp2*. Both overexpression of *admp1* and *admp2* strongly perturbed the expression of D/V marker genes. Following overexpression of *admp1*, ventral dorsal and lateral ectodermal markers were downregulated at gastrula stage and *wnt5* expression was radialized. Embryos overexpressing *admp2* also showed a strong reduction of the expression of lateral ectodermal markers but the expression of *tbx2/3* in the PMCs and of *wnt5* in the vegetal ectoderm was radialized. Also note the significant recovery of the expression of *nodal* and *chordin* at gastrula stage in embryos overexpressing *admp2*.

Supplementary Figure 5.

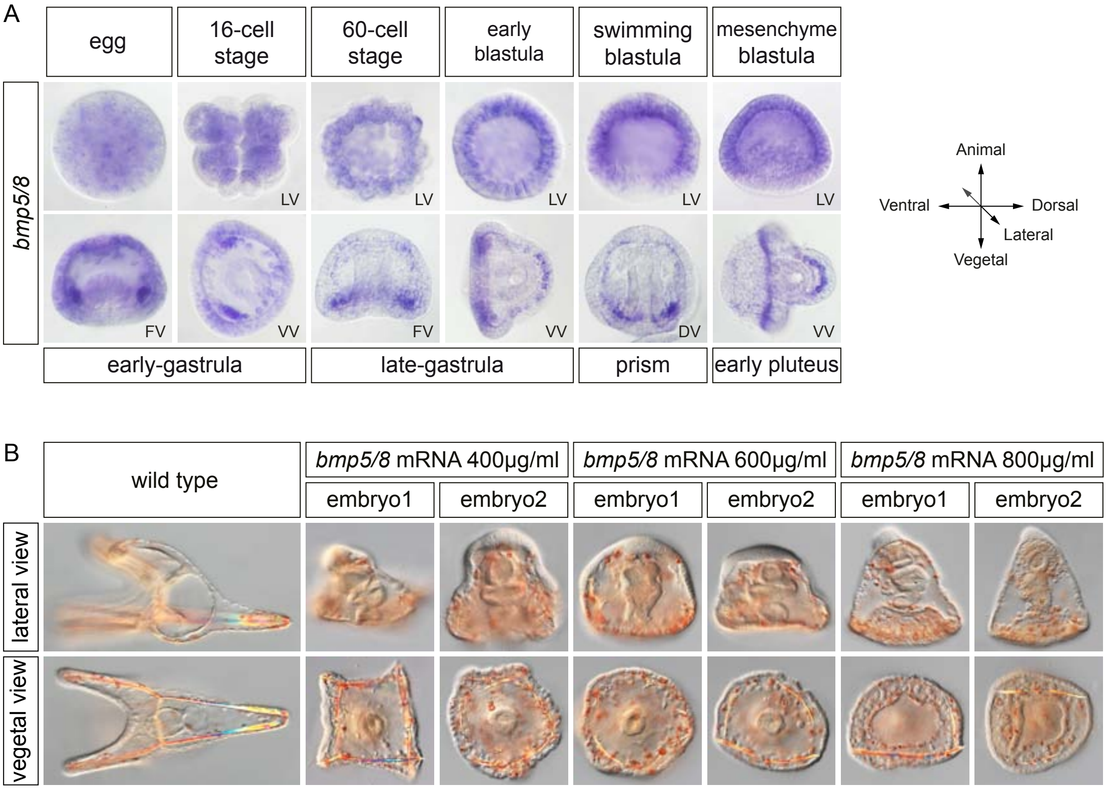

Expression pattern of *bmp5/8* and morphological phenotypes resulting from overexpression of *bmp5/8*.

A. Expression pattern of *bmp5/8*. Maternal *bmp5/8* transcripts are distributed uniformly in the egg and in embryos at cleavage stages. At swimming and mesenchyme blastula stages, zygotic transcripts accumulate in the ectoderm. During gastrulation, *bmp5/8* starts to be expressed in the PMCs located in the lateral clusters and in the dorsal chain of PMCs. At prism stage, expression of *bmp5/8* switches to the dorsal side and is restricted to the PMCs located in the apex of the larva. This late expression of *bmp5/8* is identical to that of *bmp2/4* at a similar stage.

B. Phenotypes resulting from overexpression of *bmp5/8.* Embryos overexpressing *bmp5/8* at 400 and 600 µg/ml display a Nodal loss of function-like phenotype characterized by an a strong radialization, a straight gut, a lack of mouth, and an expansion of the ciliary band to most of the ectoderm. At higher doses however, *bmp5/8* dorsalises the ectoderm resulting in a phenotype indistinguishable from that resulting from overexpression of *bmp2/4,* characterized by an elongated shape of the larva, a thin squamous ectoderm covering the embryo and the presence of long and straight spicules in the vegetal pole region.

Supplementary methods

Accession numbers for the sequences used in the trees are: Acrdi_GDF5-like (BAQ19089), Acrmi_Dpp (AAM54049), Ampfi_Afuni (AAX54512), Ampqu_PAC_15710663/BMP4-like (XP_011406805), Ampqu_PAC_15723600/BMP2-like (XP_011402506), Ancca_TGFb (AAX36083), Anoga_Mvri (AAT07309), Anoga_Myogl

(AAT07311), Apido_LOC102676483 (XP_006621960), Apifl_LOC100871171/BMP2-like (XP_012342257), Apime_60A (XP_394252), Apime_Actv (XP_001122210), Apime_LOC408789 (XP_006567370), Aplca_BMP5-like (XP_005112290), Brabe-Nodal (BAC82629), Brafl_BRAFLDRAFT_80287 (XP_002604737), Brafl_Dpp (AAC97488), Braja_BMP3/BMP3b (ACF93445), Brupa_TGFb (AAB65415), Capte_CAPTEDRAFT_110325/Nodal (ELT90528), Capte_CAPTEDRAFT_172350 (ELU11942), Capte_CAPTEDRAFT_173895 (ELU12862), Capte_CAPTEDRAFT_184506 (ELU02050), Capte_CAPTEDRAFT_184704 (ELU18783), Cioin_ADMP (BAE06303), Cioin_TGFbLig (BAE06534), Conlo_BMP2/4 (ACG70809), Cragi_BMP2-B (XP_011431554), Cragi_BMP3-like (XP_011415620), Cragi_EKC42418/BMP3-like (EKC42418), Cragi_EKC18750/BMP2 (EKC18750), Cragi_GDF2 (CAD67714), Cragi_GDF3 (CAD67715), Cragi_Nodal-like (XP_011447085), Danre_ActBa (AAH66402), Danre_ADMP (NP_571951), Danre_BMP11 (AAN03678), Danre_BMP15 (NP_001018320), Danre_BMP2b (BAA24406), Danre_BMP4 (AAC60285), Danre_BMP5 (AAH54647), Danre_BMP7 (NP_571396), Danre_Cyc (AAC34361), Danre_Lefty1 (NP_571035), Danre_MSTN/GDF8 (O42222), Danre_MSTN2 (AAT95431), Danre_Sqt (AAC34360), Danre_Vg1 (NP_571023), Dappu_DAPPUDRAFT_225730 (EFX77345), Dappu_Dpp-like (EFX74191), Drome_Actv (O61643), Drome_CG16987PA/Dawdle (AAF51204), Drome_Dpp (P07713), Drome_Gbb/60A (P27091), Drome_Mvri (NP_524626), Drome_Scw (P54631), Dugja_BMP (BAA32087), Echgr_ADMP1a (CDS21402), Galga_ADMP (NP_990153), Galga_BMP9/Dorsalin (P34822), Galga_MSTN (O42220), Galga_TGFb2 (P30371), Galga_TGFb3 (P16047), Halro_Dpp (BAA31132), Helro_HELRODRAFT_98091 (XP_009013695), Helro_P15496/HELRODRAFT_154960 (XP_009021433), Helro_P83599/Dpp (XP_009021901), Hofmi_ADMP (AID23676), Homsa_ActvBb (P09529), Homsa_BMP11 (NP_005802), Homsa_BMP15 (NP_005439), Homsa_BMP2 (P12643), Homsa_BMP3 (P12645), Homsa_BMP3b (P55107), Homsa_BMP4 (P12644), Homsa_BMP5 (P22003), Homsa_BMP6 (P22004), Homsa_BMP7 (P18075), Homsa_BMP8a (NP_861525), Homsa_BMP9/GDF2 (Q9UK05), Homsa_GDF3 (Q9NR23), Homsa_GDF5 (P43026), Homsa_GDF8 (O14793), Homsa_LeftyA (O00292), Homsa_Nodal (AAH33585), Homsa_TGFb2 (P61812), Homsa_TGFb3 (P10600), Hydvu_BMP58 (AAS01764), Hymmi_ADMP1a (CDS29508), Ixosc_B7PSK4 (/Univin-putative (XP_002402702), Ixosc_Univin-like (XP_002402701), Lotgi_BMP5-8 (BAQ19194), Lotgi_LOTGIDRAFT_110168 (XP_009045543), Lotgi_P205842/LOTGIDRAFT_205842 (XP_009063729), Lyghe_BMP3 (A0A0A9YL97), Macli_RNA1310_15976.1 (RNA1310_15976.1), Metoc_BMP7-like (XP_003737033), Mnele_TGFb (AEP16388), Musmu_ActvBa (Q04998), Musmu_BMP11 (Q9Z1W4), Musmu_BMP15 (Q9Z0L4), Musmu_BMP2 (P21274), Musmu_BMP3 (Q8BHE5), Musmu_BMP3b (NP_665684), Musmu_BMP4 (P21275), Musmu_BMP5 (NP_031581), Musmu_BMP6 (P20722), Musmu_BMP7 (P23359), Musmu_BMP8a (P34821), Musmu_BMP9/GDF2 (Q9WV56), Musmu_GDF1 (AAH79555), Musmu_GDF3 (NP_032134), Musmu_GDF5 (P43027), Musmu_GDF6 (P43028), Musmu_GDF8 (O08689), Musmu_GDNF (P48540), Musmu_Nodal (P43021), Musmu_TGFb1 (P04202), Musmu_TGFb3 (P17125), Nasvi_ADMP (NP_001288292), Nemve_Activin (ABF61781), Nemve_admp-related (AFP87424), Nemve_BMP58 (ABC88372), Nemve_Dpp (AAR27580), Nemve_GDF5 (AAS77520), Nemve_MSTNA (AGL96595), Parli_Actv (ACZ60068), Parli_ADMP1 (KP968256), Parli_ADMP2 (KT276376), Parli_BMP2/4 (ABG00199), Parli_BMP58 (KT276377), Parli_Panda (AIF71192), Parli_Lefty (AAS00535), Parli_Nodal (AAS00534), Parli_Univin (ABG00200), Patvu_BMP24 (AAM33143), Pladu_Dpp (CAJ38807), Ptyfl_ADMP1 (AJS19024), Ptyfl_ADMP2

(AJS19025), Ptyfl_BMP2/4 (BAA89012), Sacko_ADMP1 (NP_001158394), Sacko_ADMP2 (NP_001161498), Sacko_BMP3 (XP_002735398), Sacko_Nodal-A (ACY92597), Sacko_Nodal-C (NP_001164721), Scham_Dpp (AAA81169), Schma_BMP-like (ACG60602), Schme_ADMP1a (ADW27179), Schme_ADMP1b (ADW27180), Schme_ADMP1c (ADW27181), Schme_ADMP1d (ADW27182), Stemi_BMP-2 (KFM79689), Stemi_BMP3b (KFM74932), Stemi_BMP4 (KFM66469), Strpu_Activin (XP_011670199), Strpu_ADMP1 (XP_011674487), Strpu_ADMP2 (XP_003730101), Strpu_BMP24 (NP_001116977), Strpu_BMP3b (XP_786367), Strpu_BMP58 (P48969), Strpu_Panda (XP_011674529), Strpu_Lefty (NP_001123281), Strpu_MSTNA/MSTN176 (XP_789990), Strpu_MSTNB/MSTN027 (XP_003730703), Strpu_MSTNC/MSTN220 (XP_788027), Strpu_Nodal (NP_001091919), Strpu_TGFb2 (XP_793246), Strpu_Univin (P48970), Takru-MSTN1 (NP_001027843), Takru-MSTN2 (NP_001027844), Triad_P57057/TRIADDRAFT_57057 (XP_002113173), Triad_TRIADDRAFT_58663 (XP_002114631), Trica_Dpp (Q26974), Trisp_TGFB (XP_003379082), Xenla_ADMP (AAC59736), Xenla_Antiv (AAG35771), Xenla_BMP3 (Q7T2X7), Xenla_BMP3b (Q7T2X6), Xenla_BMP4 (P30885), Xenla_GDF3 (AAH73508), Xenla_GDF5 (AAT99303), Xenla_nr2 (AAA97393), Xenla_nr5 (BAB18971), Xenla_TGF-b1 (P16176), Xenla_TGFb2 (P17247), Xenla_Vg1 (P09534), Xenla-BMP2A (P25703), Xentr_BMP7 (AAT72008), Zoone_BMP3 (KDR18230), Zoone_BMP7 (KDR13694). All accession numbers are Genbank numbers except for "Lyghe_BMP3" which is a Uniprot accession number and for "Macli_RNA1310_15976.1" which is a sequence found by blast search on the *Macrostomum lignano* genome website (www.macgenome.org).

Phylogenetic analysis

Sequences were retrieved from various databases using blast search or keyword search, and aligned using Clustal Omega [1] (www.clustal.org/omega/) with default parameters. Alignment was manually checked for obvious errors using Aliview [2] (www.ormbunkar.se/aliview/) then trimmed using Trimalv1.3 [3] (trimal.cgenomics.org/) with user defined parameters (Min. percentage position to conserve: 18, Gap threshold: 0.7, Similarity threshold: 0, Window size: 1). Bayesian phylogenetic analysis was done using MrBayes 3.2.5 [4], mrbayes.sourceforge.net/) with a mixed amino-acid substitution model, and 5 millions generations. Consensus tree was generated after discarding 25% generations as burn-in.

Supplementary References

1. Sievers, F. *et al.* Fast, scalable generation of high-quality protein multiple sequence alignments using Clustal Omega. *Mol Syst Biol* **7**, 539, doi:msb201175 [pii] 10.1038/msb.2011.75.
2. Larsson, A. AliView: a fast and lightweight alignment viewer and editor for large datasets. *Bioinformatics* **30**, 3276-3278, doi:btu531 [pii] 10.1093/bioinformatics/btu531.
3. Capella-Gutierrez, S., Silla-Martinez, J. M. & Gabaldon, T. trimAl: a tool for automated alignment trimming in large-scale phylogenetic analyses. *Bioinformatics* **25**, 1972-1973, doi:btp348 [pii] 10.1093/bioinformatics/btp348 (2009).
4. Ronquist, F. & Deans, A. R. Bayesian phylogenetics and its influence on insect systematics. *Annu Rev Entomol* **55**, 189-206, doi:10.1146/annurev.ento.54.110807.090529 (2010).
